# Comparison of analytical sensitivity and efficiency for SARS-CoV-2 primer sets by TaqMan-based and SYBR Green-based RT-qPCR

**DOI:** 10.1007/s00253-022-11822-4

**Published:** 2022-02-26

**Authors:** Yile Tao, Yang Yue, Guangyu Qiu, Zheng Ji, Martin Spillman, Zhibo Gai, Qingfa Chen, Michel Bielecki, Michael Huber, Alexandra Trkola, Qiyuan Wang, Junji Cao, Jing Wang

**Affiliations:** 1 Institute of Environmental Engineering, ETH Zurich, 8093 Zurich, Switzerland; 2 Laboratory for Advanced Analytical Technologies, Empa, Swiss Federal Laboratories for Materials Science and Technology, 8600 Dübendorf, Switzerland; 3 School of Geography and Tourism, Shaanxi Normal University, Xi’an, 710119 China; 4 Department of Clinical Pharmacology and Toxicology, University Hospital Zurich, University of Zurich, 8091 Zurich, Switzerland; 5 Institute for Tissue Engineering and Regenerative Medicine, Liaocheng University, Liaocheng, 252000 China; 6 Epidemiology, Biostatistics and Prevention Institute, University of Zurich, 8091 Zurich, Switzerland; 7 Institute of Medical Virology, University of Zurich, 8057 Zurich, Switzerland; 8 Key Laboratory of Aerosol Chemistry and Physics, State Key Laboratory of Loess and Quaternary Geology, Institute of Earth Environment, Chinese Academy of Sciences, Xi’an, 710061 China; 9 CAS Center for Excellence in Quaternary Science and Global Change, Xi’an, 710061 China

**Keywords:** SARS-CoV-2, COVID-19, SYBR Green, TaqMan probe, RT-qPCR

## Abstract

**Abstract:**

The pandemic of coronavirus disease 2019 (COVID-19) continues to threaten public health. For developing countries where vaccines are still in shortage, cheaper alternative molecular methods for SARS-CoV-2 identification can be crucial to prevent the next wave. Therefore, 14 primer sets recommended by the World Health Organization (WHO) was evaluated on testing both clinical patient and environmental samples with the gold standard diagnosis method, TaqMan-based RT-qPCR, and a cheaper alternative method, SYBR Green-based RT-qPCR. Using suitable primer sets, such as ORF1ab, 2019_nCoV_N1 and 2019_nCoV_N3, the performance of the SYBR Green approach was comparable or better than the TaqMan approach, even when considering the newly dominating or emerging variants, including Delta, Eta, Kappa, Lambda, Mu, and Omicron. ORF1ab and 2019_nCoV_N3 were the best combination for sensitive and reliable SARS-CoV-2 molecular diagnostics due to their high sensitivity, specificity, and broad accessibility.

**Key points:**

*• With suitable primer sets, the SYBR Green method performs better than the TaqMan one.*

*• With suitable primer sets, both methods should still detect the new variants well.*

*• ORF1ab and 2019_nCoV_N3 were the best combination for SARS-CoV-2 detection.*

**Supplementary Information:**

The online version contains supplementary material available at 10.1007/s00253-022-11822-4.

## Introduction

After first being reported in Wuhan, China, in December, 2019, the pandemic of coronavirus disease 2019 (COVID-19) caused by an enveloped, single-stranded, positive-sense RNA betacoronavirus, severe acute respiratory syndrome coronavirus-2 (SARS-CoV-2) has already lasted for more than 1.5 years. Now, clearly, mitigation approaches (e.g., promotion of hygiene, social distancing, isolation of infected people, and restricting traveling) are crucial, but not enough for several reasons: first, unlike its close relative, severe acute respiratory syndrome coronavirus (SARS-CoV) (Lu et al. 2020), its transmission can occur during its possibly quite long prodromal period when those infected are mildly ill and carry on usual activities (Heymann et al. 2020; Zou et al. 2020). In addition, although most patients develop pneumonia and exhibit symptoms (Huang et al. 2020), many stay asymptomatic throughout the whole illness (Kronbichler et al. 2020), while still contaminating the environment (Wei et al. 2020) and causing transmissions (Hu et al. 2020). Third, vaccine breakthrough infections have been repeatedly reported (Gomez et al. 2021; Hacisuleyman et al. 2021; Luchsinger and Hillyer 2021). Moreover, when infected with the Delta variant, vaccinated people can carry and spread virus as efficiently as the unvaccinated can (Subbaraman 2021). Therefore, testing for SARS-CoV-2 infection in the whole population is central to track the spread of disease as well as to inform public policies. Yet, this may still be insufficient.

Firstly, many cases of various infected animals with evidences of both human-to-animal (Mattson 2021; McAloose et al. 2020; Munnink et al. 2021; Sit et al. 2020) and animal-to-human (Munnink et al. 2021) transmission have been reported, suggesting testing and control of SARS-CoV-2 in animals are necessary both to protect endangered animals and to control the pandemic.

Secondly, environment-to-human transmission has also been proved possible. In countries well containing the outbreak, such as China, sporadic outbreaks still occurred and were associated with international freight transportation, especially cold chain transportation (Feng et al. 2021; Pang et al. 2020; Yang et al. 2021). Moreover, poor ventilation, air conditioning (Morawska et al. 2020), and wastewater plumbing system (Gormley et al. 2020; Panchal et al. 2021) may increase transmission. On the other hand, measuring SARS-CoV-2 in indoor air and wastewater could be a sensitive tool to monitor the circulation of the virus in certain population (Medema et al. 2020). Thus, testing and monitoring SARS-CoV-2 in environments is necessary as well.

Currently, SARS-CoV-2 nucleic acid detection methods based on polymerase chain reaction (PCR) are widely accepted as the most specific, sensitive, and reliable tools for diagnosis of the infection (Petrillo et al. 2020). The TaqMan-based reverse transcription-quantitative polymerase chain reaction (RT-qPCR) has been recommended by the World Health Organization (WHO) as the gold standard method (Falzone et al. 2020). However, for developing countries deeply bogged down in the pandemic, the testing capacity of this method is still limited by the high cost and the lack of supplies and infrastructure, especially since TaqMan probes can be about ten times more expensive than simple primers due to the costly modification of dyes on both ends. SYBR Green-based qPCR may be an alternative method with lower cost and better availability of reagents. As one of the cheapest and the most widely used nucleic acid dyes (Gudnason et al. 2007), SYBR Green I emits fluorescence when bound to the minor groove of double-stranded DNA. Although unspecific amplification of non-target sequences also produces a signal, as long as their melting temperature (*T*_m_) is different from the target’s, they can be differentiated using their different melting curves (Hanna et al. 2005; Okubara et al. 2005). Thus, the choice of primers is essential for SYBR Green-based qPCR (more details of TaqMan and SYBR Green-based methods are in Supplemental Information (SI) Introduction) The WHO recommended 14 primer and probe sets developed by United States Center for Disease Control and Prevention (US CDC, USA), Charité Institute of Virology, Universitätsmedizin Berlin (Charité, Germany) (Corman et al. 2020), Institut Pasteur, Paris (IP, France), China CDC (China), the University of Hong Kong (HKU, China), National Institute of Infectious Diseases in Japan (Japan NIID, Japan), and National Institute of Health in Thailand (Thailand NIH, Thailand) (WHO 2020). Most of them target the gene coding for the Nucleocapsid protein (N) (WHO 2020), one of the four structural proteins of SARS-CoV-2 (the other three being Spike surface glycoprotein (S), Membrane (M), and Envelope (E) protein) (Chan et al. 2020). Most other sets target one of 6–11 open reading frames (ORFs) (WHO 2020). Among all ORFs, ORF1ab is the most widely used target for PCR detection, constitutes about two-thirds of the whole SARS-CoV-2 genome length, and encodes a total of 16 nonstructural proteins (nsp) including RNA-dependent RNA polymerase (RdRp) (Chan et al. 2020; Sawicki et al. 2007; van Kasteren et al. 2020). Up to now, SYBR Green method has merely been explored on patient samples with three primer sets developed by Charité (Corman et al. 2020) and one primer set developed by US CDC (Dorlass et al. 2020; Toptan et al. 2020; WHO 2020). Therefore, this study is aimed at comparing the analytical sensitivity and efficiencies of selected primer sets in TaqMan-based and SYBR Green-based RT-qPCR methods applied to 23 patient samples which included two samples of the B.1.351 lineage (Beta variant), as well as a lab cultured sample of Human Coronavirus-229E (HCoV-229E) and two environmental aerosol samples collected in Wuhan before the lockdown. We validated our qPCR methods by comparing our Ct values on these patient samples and those from certified diagnostic tests performed by the hospital. Due to the difficulties to obtain samples with virus variants, we further obtained the genetic information of six other variants, including the Delta and Omicron variants, the newly worldwide dominant ones listed as variants of concern (VOC), the Eta, Kappa, the newly found Lambda, and Mu variant listed as variants of interest (VOI) all by WHO (WHO 2021) from National Center for Biotechnology Information (NCBI) database to evaluate the sequence variability within the studied primer and probe target regions of SARS-CoV-2 genome to discuss the applicability of the SYBR Green method.

## Materials and methods

### Sample collection

Five sample sets were used in our analyses. First, 12 positive anonymized leftover clinical nasopharyngeal swab samples and five negative ones were provided by the Institute of Medical Virology, University of Zürich, Switzerland. Their Ct values were tested with the Roche cobas® SARS-CoV-2 Test (Roche, Rotkreuz, Switzerland) targeting E (SI Table S1) and ORF1 genes on a cobas 6800 (Roche, Rotkreuz, Switzerland). Second, four positive DNA samples via reverse-transcription from RNA were extracted from clinical respiratory nasopharyngeal swabs of four patients offered by Liaocheng University Hospital, China. Their Ct values were directly measured with ABI 7500 (Thermal Fisher, Waltham, USA) and Multiple Real-Time PCR Kit for detection of 2019-nCoV (XABT, Beijing, China) targeting ORF1ab and N genes (SI Table S1). Third, we collected two nasopharyngeal swabs of a volunteer diagnosed with the B.1.351 variant by sequencing. Fourth, two PM2.5 (airborne particulate matter with diameters less than 2.5 µm) samples were collected in China University of Geosciences (114.37 N, 30.54 E) in Wuhan on the 11th and 17th of January, 2020, after the outbreak and before the lockdown, using a high-volume air sampler (Tisch Environ-mental, Inc., Cleves, USA) with a flow rate of 1.13 m^3^/min and 8 × 10 inches Pallflex® TissuquartzTM air monitoring filters (Pall, New York, USA). Fifth, to evaluate cross reactions with other coronaviruses, HCoV-229E (ATCC®VR-740TM, Manassas, USA) was propagated and titrated in MRC-5 cells (ATCC®CCL-171, Manassas, USA). All samples were stored at – 20 °C before use.

### RNA extraction, cDNA, and standard plasmid synthesis

Viral RNA samples were extracted from environmental PM2.5 samples vortexed in 1.5 mL PBS, clinical respiratory nasopharyngeal swab samples, and HCoV-229E culture, respectively, using the QIAamp Viral RNA Mini Kit (Mo Bio, Qiagen, Hilden, Germany) according to the manufacturer’s protocol. Each resulted in 60 μL RNA solution; and 8 μL of it was used to synthesize cDNA with SuperScript™ III First-Strand Synthesis SuperMix for RT-qPCR (Thermal Fisher, Waltham, USA) and random hexamers.

The target genes in the first and fifth sample sets were initially detected by PCR with RedTM Imaging System (Alpha Innotech, Kasendorf, Germany) (SI Fig. S1), and the products were used for making standard plasmids utilized in qPCR. The PCR was performed on a CFX96 TouchTM Real-Time PCR Detection System (Bio-Rad, Hercules, USA) with all 14 primer sets recommended by WHO (2020) (SI Table S2), except E_Sarbeco because it cannot differentiate SARS-CoV-2 from SARS-CoV-1(Corman et al. 2020) and has been explored with SYBR Green method (Dorlass et al. 2020). Details of the reaction mixture, the thermocycler protocol, and standard plasmid synthesis are in SI Materials and Methods.

### Real-time quantitative PCR

All samples were quantified by qPCR on a CFX96 TouchTM Real-Time PCR Detection System (BioRad, Hercules, USA). To determine the analytical sensitivity of the primer sets, the second and third sets of the samples were made into 4 series of tenfold dilutions, while the standard plasmids were made into 10 series of tenfold dilutions. Details of the reaction mixture and the thermocycler protocol for both SYBR Green and TaqMan approaches are in SI Materials and Methods. All measurements were conducted in triplicates. The copy amount of each target gene was calculated based on the corresponding calibration curve obtained with the tenfold serial dilutions with the standard plasmids.

### Statistical analyses

**Fig. 1:**
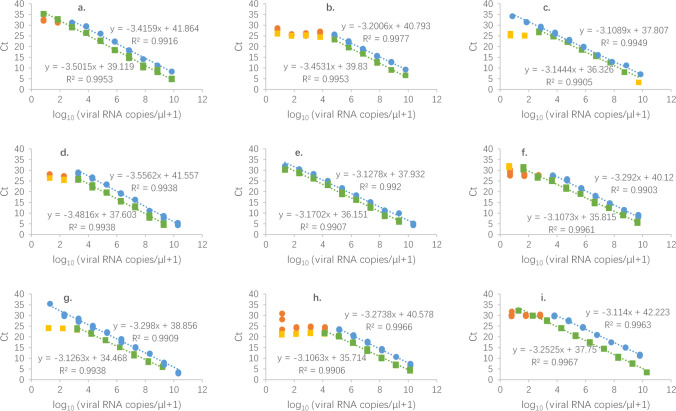
Comparisons of standard curves of the nine primer sets with TaqMan-based and SYBR Green-based RT-qPCR using standard plasmids (blue circle: TaqMan-based RT-qPCR results included in linear regression; orange circle: TaqMan-based RT-qPCR results excluded in linear regression; green square: SYBR Green-based RT-qPCR included in linear regression; yellow square: SYBR Green-based RT-qPCR results excluded in linear regression).  **a** ORF1ab, **b** N, **c** nCoV_IP4, **d **2019-nCoV_N1, **e **2019-nCoV_N3, **f **NIID_2019-nCoV_2, **g **ORF1b-nsp14, **h **HKU-N, **i** WH-NIC N

**Fig. 2:**
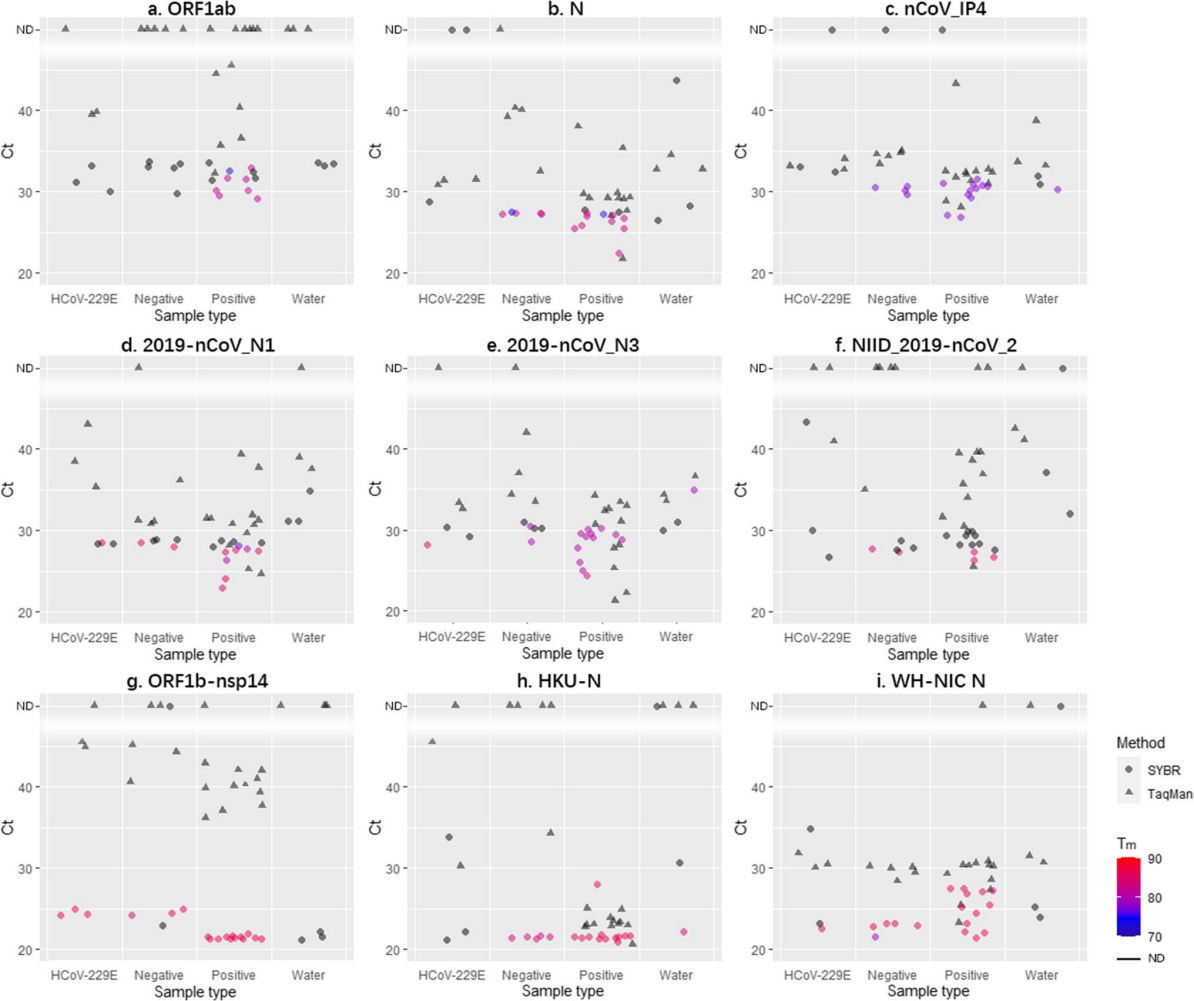
Comparisons of analytical sensitivity of the nine primer sets with TaqMan-based and SYBR Green-based RT-qPCR using SARS-CoV-2 positive, negative nasopharyngeal swabs, HCoV-229E samples, and pure water (all samples were measured in triplicates. ND: not detected. The color of the points represents the *T*_m_ of SYBR Green-based RT-qPCR products)

**Fig. 3:**
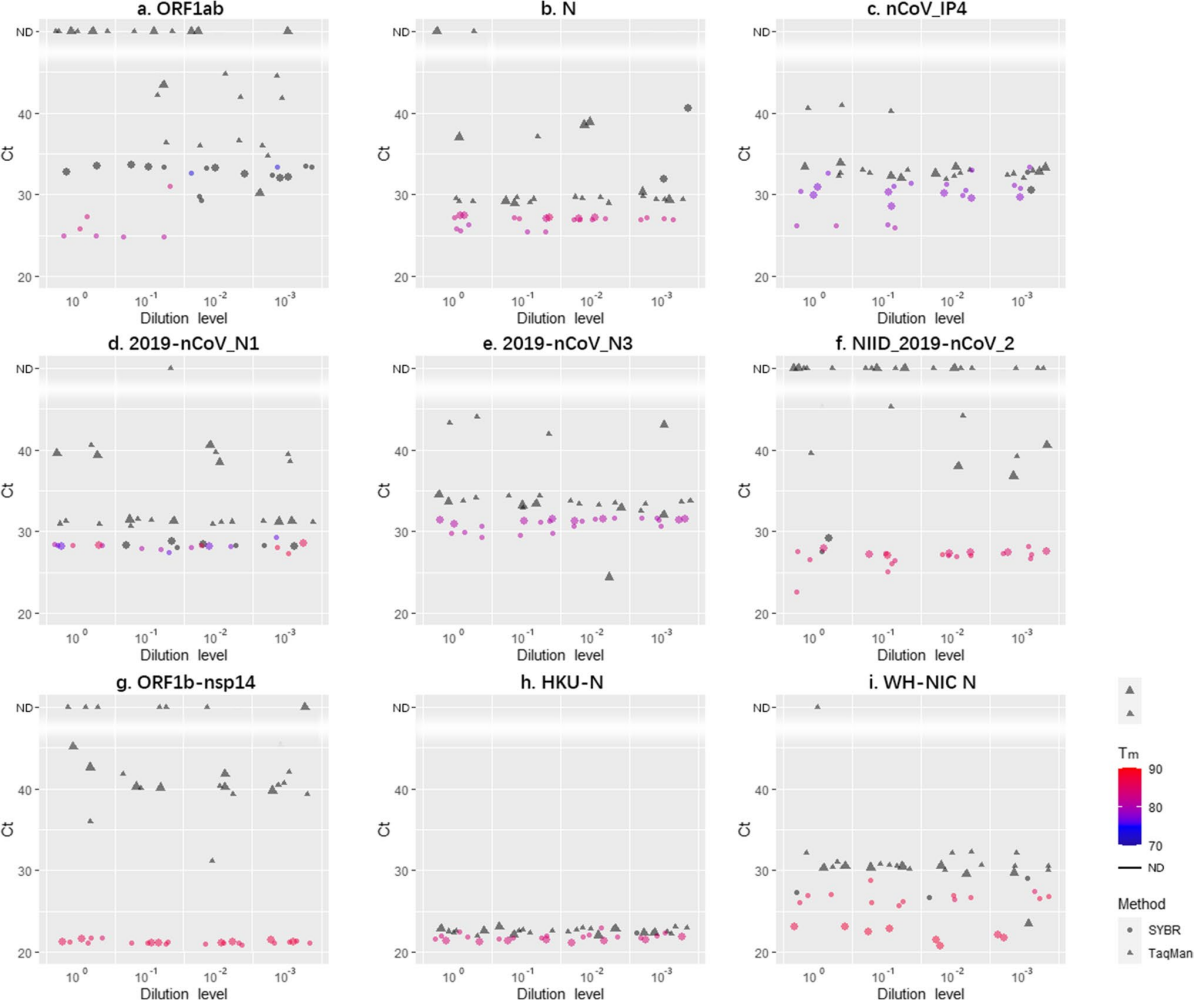
Comparisons of analytical sensitivity of the nine primer sets with TaqMan-based and SYBR Green-based RT-qPCR using SARS-CoV-2 positive nasopharyngeal swabs from 3 patients including one infected by B.1.351 lineage (all samples were separately diluted for 3 times into 4 series of tenfold dilutions. All were measured in replicates. ND: not detected. The color of the points represents the *T*_m_ of SYBR Green-based RT-qPCR products)

**Fig. 4:**
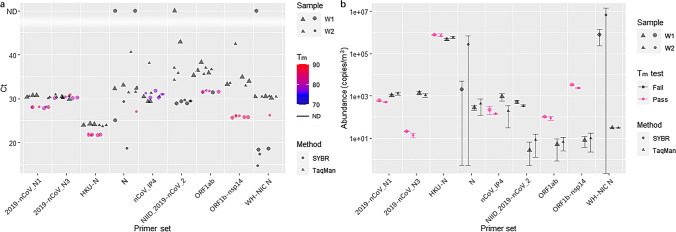
Comparisons of analytical sensitivity of the nine primer sets with TaqMan-based and SYBR Green-based RT-qPCR using aerosol samples collected in Wuhan (**a** Ct values. In this panel, the color of the points represent the *T*_m_ of SYBR Green-based RT-qPCR products. ND: not detected. **b** Absolute abundances in air. In both panels, W1: Wuhan aerosol sample obtained on the 11th of January, 2020; W2: Wuhan aerosol sample obtained on the 17th of January, 2020)

Calculation of the average values and standard deviations of qPCR data and the linear regression of the standard curves for qPCR were conducted, and Fig. 1 was drawn with Microsoft Excel 2016 (Microsoft, Redmond, USA). The analytical efficiency (*E*) of RT-qPCR assays tested with the corresponding standard plasmids was calculated using the following formula in SI. Other statistical analyses were conducted and Figs. 2, 3, and 4; SI Fig. S2, S3 were drawn in Rstudio (v.0.99.903, Rstudio PBC, Boston, USA) with more details in SI Materials and Methods.

### Identification of nucleotide mismatches of six variants at the primer and probe binding sites

Details of the downloaded sequences of Delta, Eta, Kappa, Lambda, Mu, and Omicron variants from NCBI databases are in SI Materials and Methods. Every 400 sequences of the same variant were aligned directly on NCBI and downloaded until there were less than 400 sequences in the variant group which were aligned together and downloaded. The quality of the multiple sequence alignment (MSA) results was checked in AliView (Larsson 2014). Edits to the alignment were manually introduced when necessary to obtain the best alignment. The sites bound by the same primer or probe binding site were copied and pasted into one FASTA file. 42 FASTA files were generated and then uploaded separately to SequenceTracer for sequence stratification (http://www1.szu.cz:8080/EntropyCalcWeb/sequences) according to Khan’s protocol (Khan and Cheung 2020a).

## Results

### Comparisons of RT-qPCR primer sets with different approaches on standard plasmids

After gel electrophoresis (SI Fig. S1) and blue-white screening (SI Fig. S2), nine primer sets’ standard plasmids were successfully made according to the sequencing results. PCR amplification efficiencies of each primer set using tenfold dilutions of the respective plasmids (Fig. 1) were all above 90% and below 110% (SI Fig. S3a). ORF1ab, N, WH-NIC N, and nCoV_IP4’s amplification efficiencies of the TaqMan approach were higher than the SYBR Green ones, while the other primer sets differed, yet all matched the criteria for efficient RT-qPCR (Vogels et al. 2020). To measure the analytical sensitivity, we used the Ct value with which the linear regression of the dilution series would cross the *y*-intercept when tested with one viral RNA copy/μL. All measured sensitivities (*y*-intercept Ct values) were around 40 Ct, and the SYBR Green ones were significantly lower than the TaqMan ones (*p* = 0.00097). Thus, under ideal conditions, the SYBR Green approach should be more sensitive. However, for some primer sets, the Ct value curves would plateau with low viral load, not correlated to *y*-intercept Ct (Fig. 1). In this situation, N and HKU-N were quite inaccurate for both methods, while the TaqMan method was more accurate to quantify samples with nCov_IP4 and ORF1b-nsp14, and the SYBR Green method offered better accuracy with ORF1ab, NIID_2019-nCoV_2, and WH-NIC N. Both methods were equally accurate with 2019-nCoV_N3.

### Comparisons of RT-qPCR primer sets with different approaches on clinical and HCoV-229E laboratory samples

Although the Ct values of most samples measured by SYBR Green method were detected and lower than 40 in our settings, the signal peaks indicating melting were often undetected in the melting curves (Fig. 2), allowing for easy identification of false positive samples. With TaqMan approach, a result was considered positive as long as the Ct value was less than 40. In our experiments, negative samples consisted of ddH_2_O samples, HCoV-229E samples extracted from laboratory culture, and negative clinical samples.

For ddH_2_O samples, all primer sets worked well for the SYBR Green method, but only ORF1ab, ORF1b-nsp14, and HKU-N worked well for both methods (Fig. 2a, g, h). For HCoV-229E samples, N, nCoV_IP4, and WH-NIC N showed positive results with the TaqMan-based method, but negative with the SYBR Green-based method (Fig. 2b, c, i), while ORF1b-nsp14 performed reversely (Fig. 2g). These 4 primer sets and HKU-N performed poorly at differentiating negative clinical samples from positive ones with the SYBR Green-based method. N and nCoV_IP4 delivered four positive results out of five negative samples (Fig. 2b, c). Additionally, nCoV_IP4 could not distinguish positive samples from negative ones with the TaqMan-based method either. ORF1ab was the best to rule out negative samples with both methods, yielding no false-positive results (Fig. 2a).

Twelve undiluted positive clinical samples were tested (Fig. 2). NIID_2019-nCoV_2 was the worst with the SYBR Green approach with only three positive results out of the 12 positive samples (Fig. 2f). nCoV_IP4, 2019-nCoV_N3, HKU-N, and WH-NIC N assessed all positive clinical samples correctly with both methods (Fig. 2c, e, h, i). N and 2019-nCoV_N1 misidentified respectively two and four positive samples with the SYBR Green-based method, while they made no mistake with the TaqMan-based method (Fig. 2b, d). The other two sets showed fewer false-negative results with the SYBR Green-based method than with the TaqMan-based method (Fig. 2a, g).

All undiluted samples failed the normality test (SI Table S3). Further randomization test on matched samples showed that the Ct values of the SYBR Green approach were significantly lower than the TaqMan approach with almost all primers, except N (SI Table S4). Friedman test and post hoc analysis on matched undiluted samples showed a complicated result with the SYBR Green method (SI Table S5), while with the TaqMan approach, the primer sets could be divided into two groups with significantly different Ct values from each other (SI Table S6). The group with lower Ct values included nCoV_IP4, N, HKU-N, 2019-nCoV_N1, 2019-nCoV_N3, and WH-NIC N; the majority of the primer sets targeting N region implied the higher sensitivity of primer sets targeting N region. Most positive and negative undiluted samples failed the normality test (SI Table S7). The Ct values of the positive undiluted samples measured by the SYBR Green approach were only significantly lower than the negative ones for N, 2019-nCoV_N1, 2019-nCoV_N3, and ORF1b-nsp14 (SI Table S8), while two more primer sets led to significantly lower Ct for the positive samples than negative ones with the TaqMan approach (SI Table S8). Noticeably, the results of *T*_m_ test important for the SYBR Green approach could not be included into the statistical test here. Thus, the statistical test cannot suggest poor performances of the SYBR Green approach utilizing primer sets like ORF1ab which depended likely on both *T*_m_ test and Ct values. In fact, according to the results of Friedman test and post hoc analysis (SI Table S9), all Ct values measured by the SYBR Green approach were not significantly different from the ones measured by the hospital, while all the Ct values significantly different from the ones measured by the hospital were generated by the TaqMan approach, utilizing the following primer sets: nCoV_IP4 (*p* = 0.03915), NIID_2019-nCoV_2 (*p* = 0.00125), ORF1ab (*p* = 2.90 10^−5^), and ORF1b-nsp14 (*p* = 0.00019). Thus, the SYBR Green method provided Ct values closer to the ones obtained by the certified hospital diagnostic tests and could be considered as reliable in our tested cases.

For diluted positive clinical samples, only the Ct values of both methods with HKU-N fit the normal distribution (SI Table S3). The Ct values of the SYBR Green approach were significantly lower than the TaqMan approach with all primer sets (Fig. 3, SI Table S4). Friedman test and post hoc analysis showed similar but slightly more complex results of the diluted samples compared to the undiluted samples (SI Table S10, S11). For example, the Ct values of HKU-N were significantly lower than all other primer sets except N and WH-NIC N, suggesting that they were not only extremely sensitive but also unspecific. Among all the primers, 2019-nCoV_N3 and ORF1b-nsp14 delivered positive results correctly for all samples with the SYBR Green-based method (Fig. 3e, g–i). However, the Ct values of the latter set were all smaller than 30, and there was almost no difference in the Ct values among different dilutions. On the other hand, ORF1ab and N clearly had difficulties to identify low viral concentrations (Fig. 3a, b). They could not detect Beta variant either (Fig. 3a, b), though no reports showed this variant contained mutations in their target regions. Therefore, further study and evaluation are necessary to ensure the accurate diagnosis of this variant.

### Comparisons of RT-qPCR primer sets with different approaches on environmental samples

Our positive RT-qPCR results on environmental samples indicated that SARS-CoV-2 RNA existed in the aerosol of Wuhan after the outbreak and before the lockdown (Fig. 4), though the absolute abundances measured with different primer sets and methods varied from 2.66 to 3338.07 copies/m^3^ due to the limitation of RT-qPCR itself (Park et al. 2021). The ambient PM2.5 samples were collected from a university campus, not from a hospital housing COVID patients, suggesting with sensitive methods and primer sets, detection of SARS-CoV-2 in urban aerosol has the potential for COVID monitoring, early warning and infection control. NIID_2019-nCoV_2, N, and WH-NIC N failed with the SYBR Green-based method because of undetected melting peaks. All Ct values of the SYBR Green approach were either smaller or comparable to the TaqMan approach’s. Yet, after calibration with their respective standard curves, the abundances of 2019-nCoV_N1, 2019-nCoV_N3, and nCoV_IP4 with the SYBR Green approach were lower than the respective TaqMan’s. Not only were HKU-N’s Ct values of lower than 25 but also the abundances were about 3-magnitude larger than the other primer sets’, suggesting HKU-N might be easily affected by the complex contents in environmental samples and should not be used for such samples.

### Nucleotide mismatches of six variants at the primer and probe binding sites

All primer–probe sets have at least one significant detected mismatch frequency which means above the 0.5% threshold (Elaswad and Fawzy 2021; Khan and Cheung 2020b; Vogels et al. 2020) with all six variants (SI Table S12-16). nCoV_IP2 and ORF1ab both contained only one primer or probe with only one significant mismatch with only one variant (SI Table S12-15). The NIID-2019-nCoV_2 reverse primer showed a single-nucleotide mismatch (G-C) with all tested sequences (SI Table 12–16) as reported previously (Elaswad and Fawzy 2021). However, if this could be corrected, the bespoke reverse primer would only have one slightly significant mismatch (C-T) for Lambda variants. The reverse primer of RdRp-SARS and RdRp-CoV-19 and ORF1b-nsp14 showed a single-nucleotide mismatch (R-T) with all tested variants (SI Table S12-16), which may need correction. The forward primer of N had the most mismatches. It displayed one-nucleotide mismatches with more than 98% of Delta and Mu variant sequences, more than 96% Kappa variant sequences, slightly significant two-nucleotide mismatches in Kappa variant sequences, and three-nucleotide mismatches with more than 99% of Lambda variant sequences (SI Table S12, S14-16). Its lowest mismatch frequency was found in Eta variant, a one-nucleotide mismatch (T-C) in still more than 1% of sequences (SI Table S13). The probe of 2019-nCoV_N1 also had several mismatches. Most of them were only slightly significant, but one of them displayed one-nucleotide mismatches with more than 98% of Omicron variant sequence (SI Table S17).

## Discussions

Our study provides a comprehensive and independent comparison of analytical performance of different primer sets for SARS-CoV-2 with two methods tested on various samples. We found with suitable primer sets that the performance of the SYBR Green approach can be comparable or even better than the performance of the TaqMan approach, as has been reported that E_Sarbeco by Charité has comparable performances with both approaches (Corman et al. 2020; Dorlass et al. 2020).

Among all the studied primer sets, RdRp-SARS and RdRp-CoV-19 were the least recommended because of low sensitivity. Though one study suggested they were as sensitive and specific as the primers targeting ORF1ab and N, better than the ones targeting E and S (Mollaei et al. 2020), more studies differed (Nalla et al. 2020; Toptan et al. 2020; Vogels et al. 2020). Their low sensitivity may be explained by the presence of multiple wobble nucleotides (Eis-Hubinger et al. 2020; Toptan et al. 2020). Moreover, as found in this study (SI Table 12 & 14) and several previous studies (Alvarez-Diaz et al. 2020; Elaswad and Fawzy 2021; Khan and Cheung 2020b; Miranda and Weber 2021; Pillonel et al. 2020), they did not fully match the available SARS-CoV-2 genomes. Though the mismatches had little effect on their efficiencies (Corman and Drosten 2020; Nalla et al. 2020) and increased their coverage, they also increased the coverage for SARS-CoV-1 and other human coronaviruses (Miranda and Weber 2021). We found that the amplification of SARS-CoV-2 with RdRp-SARS was not affected by mismatches either.

ORF1 which included RdRp region was recommended as one of the best regions for SARS-CoV-2 identification besides the N region (Karagöz et al. 2020; Mollaei et al. 2020). Yet, positive selection has been demonstrated for specific residues of the non-structural proteins of ORF1ab and the accessory proteins ORF3a and ORF8 which might affect PCR test accuracy (Velazquez-Salinas et al. 2020).

In previous studies, among our choices, nCoV_IP4 was the only one targeting the ORF1 gene which matched perfectly with animal and human isolates (Elaswad and Fawzy 2021; Khan and Cheung 2020b). However, we found it imperfect for Delta, Kappa, and Mu variants (SI Table 12, 14, 16). Additionally, both nCoV_IP2 and nCoV_IP4 had multiple non-specific bands in PCR products in our results, which might cause nCoV_IP4’s high false-positive rate with both methods.

Though mismatches of ORF1ab was found in animal isolates (Elaswad and Fawzy 2021), up to now, it is still almost perfect for human ones according to previous studies (Alvarez-Diaz et al. 2020; Khan and Cheung 2020b) and our results (SI Table 12–16). In fact, ORF1ab was recommended as the most sensitive and reliable one repeatedly (Jung et al. 2020; Mollaei et al. 2020). So far, only one case was found to have a single substitution at the seventh probe position with no considerable effect (Alvarez-Diaz et al. 2020). We did not find this substitution with our studied variants, but slightly significant single substitution (C-T) at the second probe position only with Eta variant. Although it might not be the most sensitive probe and had troubles with identifying Beta variant, it had the lowest false-positive rate. Additionally, using SYBR Green instead of TaqMan improved its sensitivity. This performance difference may become more significant with variants since mismatches have currently only been found in its probe unnecessary for SYBR Green approach, not in its primers. On the other hand, ORF1b-nsp14 along with HKU-N should not be used with the SYBR Green approach and judiciously with the TaqMan approach. They did not align with the target genome well in a previous study (Miranda and Weber 2021) and in ours (SI Table S12-16). The 3′ end of the reverse primer of HKU-N aligned with the third codon position of its corresponding ORF which made it susceptible to false negative results facing the increasing viral genetic variability (Alvarez-Diaz et al. 2020). We did not detect this kind of synonymous mutation but found two different kinds of single substitutions with Delta, Kappa, and Lambda variants in the binding region of the reverse primer (SI Table 12, 14, 15). Additionally, they formed primer dimers, which might cause their high fake positive rate and extremely small Ct values with SYBR Green approach. Also, the probe of HKU-N was reported to be strongly different from all references by four nucleotide sites and displayed the formation of a highly stable hairpin structure and two self-dimer structures in a previous study (Alvarez-Diaz et al. 2020).

Although mismatches have been revealed in N, 2019-nCoV_N1, 2019-nCoV_N3, NIID_2019-nCoV_2, and WH-NIC N previously (Alvarez-Diaz et al. 2020; Elaswad et al. 2020; Khan and Cheung 2020b) and in this study, suggesting the N gene may be under positive selective pressure where many mutations accumulates (Elaswad et al. 2020; Lo Presti et al. 2020); the N region has been still widely considered as the best and most commonly used for SARS-CoV-2 identification (Karagöz et al. 2020; Mollaei et al. 2020).

NIID_2019-nCoV_2 was recommended as the most sensitive and reliable primer set by one previous research (Jung et al. 2020). It would have been an almost perfect match for the studied variants, if the 15th position of its reverse primer was corrected from G to C (SI Table S12-16). With the TaqMan-based method, it had a quite low false-positive rate. Yet, we do not recommend it for the SYBR Green-based method due to its high false-negative rate and failure in *T*_m_ test of environmental samples.

N and WH-NIC N also failed for environmental samples. N primer hybridization was found to be critically affected by the accumulated genetic diversity of the Colombian SARS-CoV-2 strains (Alvarez-Diaz et al. 2020) and almost all variants in our study (SI Table S12-16). Additionally, its fake positive rate was relatively high and inaccurate to measure samples with low viral loads. WH-NIC N has not been studied widely and did not merit recommendation due to high fake positive rate especially with the SYBR Green method.

2019-nCoV_N1, 2019-nCoV_N2, and 2019-nCoV_N3 were the most studied ones. One study found 2019_nCoV_N2 to be the most sensitive (Nalla et al. 2020), while another found it not as sensitive as 2019_nCoV_N1 (Vogels et al. 2020). Notwithstanding that it was almost a perfect match to most variants in our study (SI Table S12, S14, S15), our results agreed with the second study that 2019_nCoV_N2 was quite insensitive, since it is the only primer set with which we failed to obtain a positive result from PCR. Both cited studies found that 2019-nCoV_N1 and 2019-nCoV_N2 performed better than 2019_nCoV_N3 (Nalla et al. 2020; Vogels et al. 2020). A possible explanation is that both the forward and reverse primer of 2019_nCoV_N3 aligned with the third codon position of their corresponding ORFs, while among the other two, only the 3′ end of the forward primer of 2019_nCoV_N1 had the same problem, so 2019_nCoV_N3 is more likely to be susceptible to false-negative results with rising viral genetic variability (Alvarez-Diaz et al. 2020). These synonymous mutations were undetected in our study. We found 2019_nCoV_N3 was the most sensitive one in every aspect, and both false-positive rates of 2019_nCoV_N1 and 2019_nCoV_N3 were lower with SYBR Green than with TaqMan. This aligned with the fact that single mismatches in the binding regions of both probes have been detected, especially for the one of 2019_nCoV_N1 with Omicron variant (SI Table S12-17), suggesting that the SYBR Green-based method may be a better choice for these two.

To conclude (SI Table S18), RdRp-SARS, RdRp-CoV-19, N, 2019_nCoV_N2, and nCoV_IP4 did not meet performance expectations in our setting; NIID_2019-nCoV_2, ORF1b-nsp14, HKU-N, and WH-NIC N performed better with the TaqMan approach, while the SYBR Green-based method brought out the best side of ORF1ab, 2019_nCoV_N1, and 2019_nCoV_N3. Thus, we recommend these three, because (1) the SYBR Green-based method is relatively cheap, which is an attractive and important practical aspect for laboratory studies and developing countries where large-scale testing is still essential due to the local shortage of vaccines, medical facilities and infrastructure; (2) they perform well on both clinical and environmental samples; and (3) they have been widely used with the TaqMan approach around the world providing abundant accessibility for trial of both methods and comparison of the results. Among them, ORF1ab and 2019_nCoV_N3 afford the best combination for sensitive and reliable SARS-CoV-2 molecular diagnostics due to their complementary merits.

## Supplementary Information

Below is the link to the electronic supplementary material.

Supplementary file1 (PDF 2978 KB)

## Data Availability

The authors confirm that the datasets supporting the findings and conclusions of this study are available within the article and its supplementary information file.
