# Supplementary material for: Comparison of analytical sensitivity and efficiency for SARS-CoV-2 primer sets by TaqMan-based and SYBR Green-based RT-qPCR
Source: Appl Microbiol Biotechnol. 2022 Feb 26;106(5-6):2207–18. doi: 10.1007/s00253-022-11822-4 (PMC8881549; doi:10.1007/s00253-022-11822-4)
Supplement: Supplementary file 1 — Supplementary file1 (PDF 2978 KB) [file 253_2022_11822_MOESM1_ESM.pdf]

Applied Microbiology and Biotechnology

Supplementary Information

Comparison of Analytical Sensitivity and Efficiency for SARS-CoV-2 Primer Sets by TaqMan-based and SYBR Green-based RT-qPCR

Yile Tao[1, 2], Yang Yue[1, 2], Guangyu Qiu[1, 2], Zheng Ji[3], Martin Spillman[1, 2], Zhibo Gai[4], Qingfa Chen[5], Michel Bielecki[6], Michael Huber[7], Alexandra Trkola[7], Qiyuan Wang[8, 9], Junji Cao[8, 9] and Jing Wang[1,2,*]

1. Institute of Environmental Engineering, ETH Zurich, Zurich 8093, Switzerland;
2. Laboratory for Advanced Analytical Technologies, Empa, Swiss Federal Laboratories for Materials Science and Technology, Dübendorf 8600, Switzerland
3. School of Geography and Tourism, Shaanxi Normal University, Xi'an 710119, China
4. Department of Clinical Pharmacology and Toxicology, University Hospital Zurich, University of Zurich, Zurich 8091, Switzerland
5. Institute for Tissue Engineering and Regenerative Medicine, Liaocheng University, Liaocheng 252000, China
6. Epidemiology, Biostatistics and Prevention Institute, University of Zurich, Zurich 8091, Switzerland
7. Institute of Medical Virology, University of Zurich, 8057 Zurich, Switzerland
8. Key Laboratory of Aerosol Chemistry and Physics, State Key Laboratory of Loess and Quaternary Geology, Institute of Earth Environment, Chinese Academy of Sciences, Xi'an 710061, China
9. CAS Center for Excellence in Quaternary Science and Global Change, Xi'an 710061, China

*Correspondence: jing.wang@ifu.baug.ethz.ch

Abstract:

The pandemic of coronavirus disease 2019 (COVID-19) continues to threaten public health. For developing countries where vaccines are still in shortage, cheaper alternative molecular methods for SARS-CoV-2 identification can be crucial to prevent the next wave of infections. Therefore, in this study, we evaluated the 12 primer sets recommended by the World Health Organization (WHO) on testing both clinical patient and environmental samples with the gold standard diagnosis method: TaqMan-based RT-qPCR and a cheaper alternative method: SYBR Green-based RT-qPCR. We found that using suitable primer sets, such as ORF1ab, 2019_nCoV_N1 and 2019_nCoV_N3, the

performance of the SYBR Green approach was comparable or even overperformed than the TaqMan approach, even when considering the newly dominating or emerging variants, including Delta, Eta, Kappa, Lambda and Mu. ORF1ab and 2019_nCoV_N3 were found to be the best combination for sensitive and reliable SARS-CoV-2 molecular diagnostics due to their high sensitivity, specificity and broad accessibility.
Keywords: SARS-CoV-2, COVID-19, SYBR Green, TaqMan probe, RT-qPCR

Supplementary Introduction

A typical TaqMan real-time PCR assay includes a fluorogenic probe designed with a fluorescent reporter dye on the 5’ end and a quenching dye on the 3’ end. The probe anneals specifically to the PCR amplicon. The 5’ exonuclease activity of the Taq polymerase then cleaves the probe freeing the 5’ reporter dye, so the quencher dye is stopped from masking the dye’s fluorescence. The reporter dye is released during the early exponential phase of the PCR reaction, and the amplication cycle that first produces a detectable amount of dye is recorded by the optical module as the threshold cycle(Giulietti et al. 2001). The threshold cycle value (Ct) is inversely proportional to the original relative abundance of the target genes. However, TaqMan probes can be about ten times more expensive than simple primers due to the costly modification of dyes on both ends.

SYBR Green-based qPCR may be an alternative that could fulfill the urgently needed testing capacity. Its mechanism is similar to Taqman-based qPCR, but instead of probes, it uses a universal fluorescent DNA dye to quantify the amplicon product directly. Nucleic acid dyes are generally much cheaper than probes(Kubista et al. 2006), while SYBR Green I is the cheapest and the most widely used among all such dyes(Gudnason et al. 2007). It binds to the minor groove of double-stranded DNA. The fluorescence of the bound SYBR green I is about 20-fold greater than the unbound dye. Thus, it can be used with any primer pair,

amplicon, and model of thermocyclers without developing a specific probe. Although unspecific amplification of non-target sequences also produces a signal, as long as the amplified sequences do not share a similar melting temperature (T_m) with the target, they can be differentiated using their different melting curves(Hanna et al. 2005; Okubara et al. 2005). This means, the choice of primers is essential for SYBR Green-based qPCR.

Supplementary Materials and Methods

Sample details

PCR, qPCR reaction mixture, their thermocycler protocol and standard plasmid synthesis

The PCR reaction mixture was 20 µL, containing 1 µL of DNA template, 14.92 µL of double-distilled water (ddH_2O), while the volumes of other components were determined proportionally according to the protocol of *Taq* DNA Polymerase, recombinant (Life Technologies, Thermo Fisher, Waltham, USA). The thermocycler protocol was initial denaturation for 3 min at 95°C, followed by 40 cycles of 30s at 95°C, 30s at the respective melting temperatures (T_m) of the primer set (SI Table S2), and 30s at 72°C, and a final extension at 72°C for 10 min.

The PCR products were run on 2% agarose gel electrophoresis, checked with Red™ Imaging System (Alpha Innotech, Kasendorf, Germany) (SI Fig. 1), purified with a QIAquick® gel extraction kit (Qiagen, Hilden, Germany), and then cloned into *Escherichia coli* JM109 with pGEM-T Easy vector system (Promega, Madison, USA). Positive clones were randomly selected by blue-white screening method, then cultivated and checked by PCR. The plasmids were extracted with a Qiaprep spin miniprep kit (Qiagen, Hilden, Germany) and sequenced

by an ABI 3730xl DNA Analyzer (Applied Biosystems, Waltham, USA) to serve as the standard plasmids for qPCR. The concentration of the extracted plasmids was quantified by Infinite® 200 PRO plate reader (TECAN, Männedorf, Switzerland).

For SYBR Green approach, the reaction mixture of qPCR was 10 μL, containing 5 μL SsoAdvanced Universal SYBR Green supermix (BioRad, Hercules, USA), 0.25 μL of each 10μM primer, 0.5 μL of template and 4 μL of ddH2O, with the same primers and T_m as the ones for PCR. The qPCR cycler condition was almost the same as the one for PCR, just without the final extension. Purity of the products was checked using the melting curve method (T_m test). For TaqMan approach, the reaction mixture of qPCR was also 10 μL, containing 5 μL SsoAdvanced™ Universal Probes Supermix (BioRad, Hercules, USA), 0.25 μL of each 10μM primer, 0.25 μL of 10μM probe, 0.5 μL of template and 3.75 μL of ddH_2O, with the primers and extension temperatures the same as the primers and T_m for PCR and SYBR Green-based qPCR. The PCR cycler condition was initial denaturation for 1 min at 95°C, followed by 45 cycles of 10s at 95°C, 30s at the respective extension temperature of the primer set.

Statistical analyses

The analytical efficiency (E) of RT-qPCR assays tested with the corresponding standard plasmids was calculated using the following formula with consideration of the slope of the regression line:

$$\mathrm{E} = 100 \times (10^{-\frac{1}{slope}} - 1)$$

Shapiro-Wilk test was performed for normality tests of the reciprocals of all different sets of Ct values. The reciprocal was considered 0, if Ct value was not detected. For the sets which passed the normality test, Paired Student's t test was used to compare the differences

between two methods' performances. Otherwise, randomization test on matched samples was performed. Randomization test was also applied to identify the differences of both methods' performances between positive and negative samples. Friedman test was used for assessing the performances of different primer sets on clinical, HCoV-229E laboratory and ddH_2O samples. Based on the results, post-hoc analyses were further used to identify the significantly different groups from each other. Friedman test and post-hoc analysis were also used to compare the PCR results of different primer sets and methods on the clinical swab samples (the first sample set) and the results from the hospital.

Identification of nucleotide mismatches of six variants at the primer and probe binding sites

A total of 10106 SARS-CoV-2 Delta variant genomes, including 9892 of lineage B.1.617.2, 48 of lineage AY.1 or B.1.617.2.1, 166 of lineage AY.2 were downloaded from the NCBI databases as of 6 July 2021. 2041 SARS-CoV-2 Eta variant genomes (lineage B.1.525), 731 SARS-CoV-2 Kappa variant genomes (lineage B.1.617.1) and 628 SARS-CoV-2 Lambda variant genomes (lineage C.37) were downloaded on 12 July 2021. 1506 SARS-CoV-2 Mu variant genomes (lineage B.1.621) were downloaded on 8 September 2021. A total of 4588 SARS-CoV-2 Omicron variant genomes, including 18 of lineage B.1.1529 and 4570 of lineage BA.1 were downloaded from the NCBI databases as of 29 December 2021.

Supplementary Figures and Results

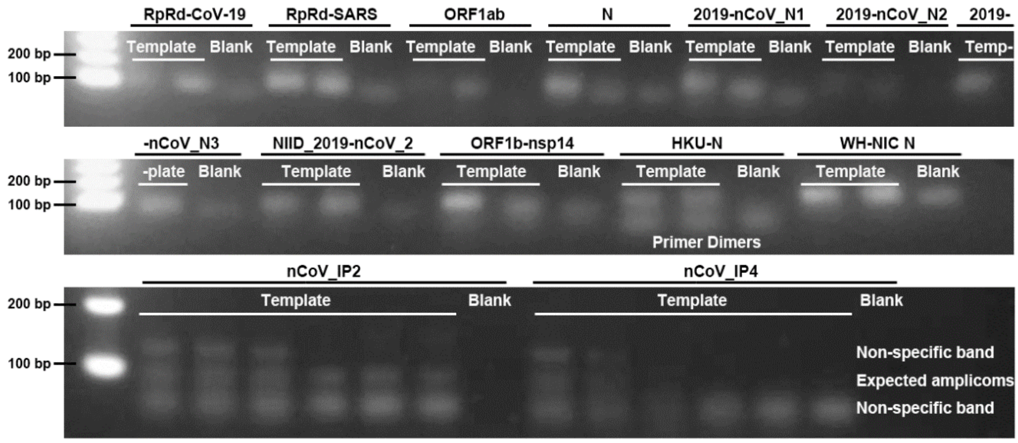

Fig. S1 Gel electrophoresis results from PCR (40 cycles) in 2% agarose gel

All the amplicons generated by the specific primer sets had similar molecular weight (Fig. S1). The bands of amplicon of 2019-nCoV_N2 primer set were very difficult to identify, suggesting this primer set was likely less sensitive than other primer sets, which was in agreement with the finding that 2019-nCoV_N2 was less sensitive than 2019-nCoV_N1 primer set (Vogels et al. 2020). Most products did not have multiple bands, except for the ones developed by HKU and IP. For the HKU ones, there were blurry primer dimer bands in the products of ORF1b-nsp 14 and clear ones in those of HKU-N, while for the IP ones, multiple non-specific bands were found. Both IP ones may possibly disturb the SYBR Green approach and cause false positive, whereas they may only decrease the PCR efficiency in the TaqMan approach. Both RdRp-CoV-19 and RdRp-SARS primer sets enable the production of amplicons, suggesting these two primer sets have difficulties to differentiate their target coronaviruses.

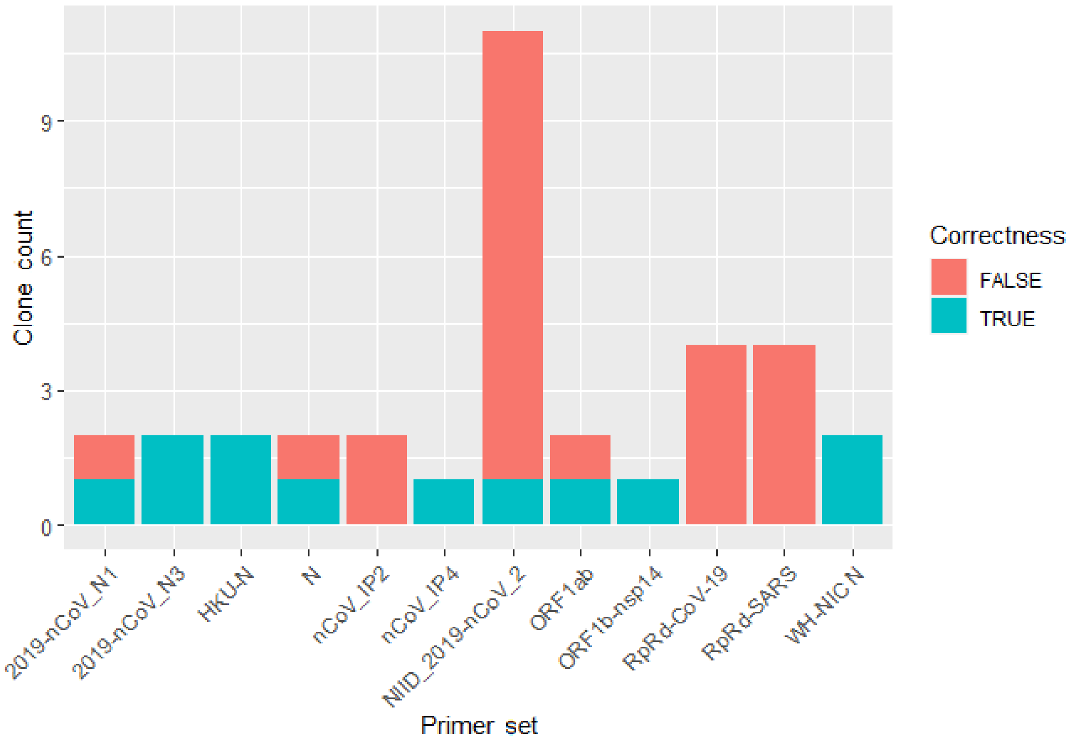

Fig. S2 Positive clones confirmed by Sanger sequencing

To further evaluate and sequence the PCR products, and synthesize the standard plasmids, blue-white screening was performed. No positive clone of 2019-nCoV_N2 was found likely due to the poor quality of its PCR product, further suggesting the insensitivity of this primer set. The positive clones of RdRp-CoV-19, RdRp-SARS, and nCoV_IP2 were all fake positive clones (SI Fig. S2) with much shorter lengths than expected true ones according to sequencing results. This was not surprising, since nCoV_IP2 had multiple non-specific bands, while RdRp-SARS assay had been confirmed as a less sensitive assay not reliable at <1,000 viral RNA copies/μl of extracted nucleic acid(Vogels et al. 2020). Therefore, the above four primer sets were Excluded from further experiments as well as E_Sarbeco, and only the performances of the other nine recommended by WHO were assessed in the following RT-qPCR experiments.

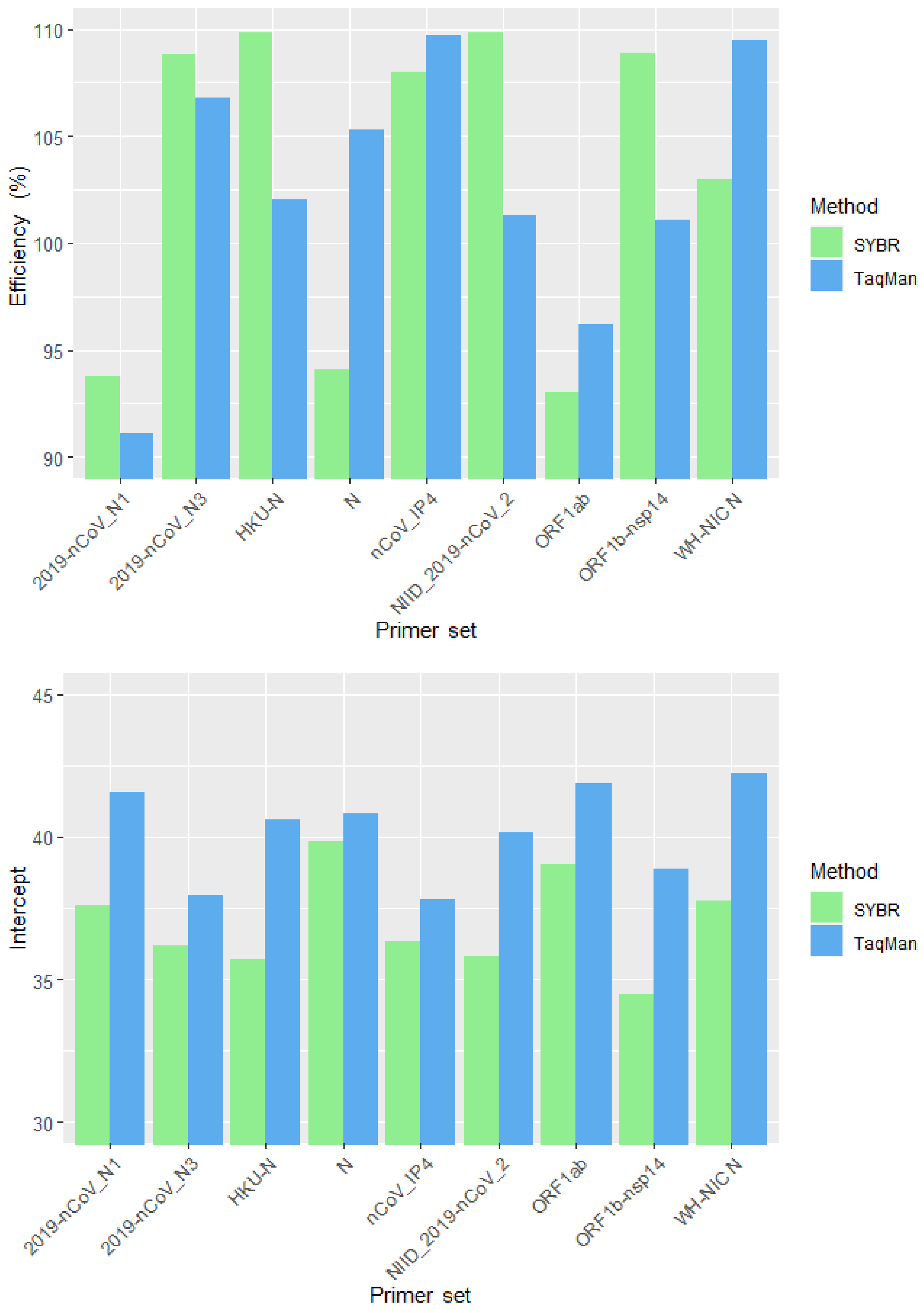

Fig. S3 Comparisons of PCR efficiency (up) and y-intercept Ct value (down) of the nine primer sets with TaqMan-based (blue) and SYBR Green-based (green) RT-qPCR using standard plasmids

Supplementary Tables

Table S1. The Ct values of two sample sets

Sample set	Sample ID	Ct	1/Ct
1[a]	UZH1	35.19	0.02842
	UZH2	33.76	0.02962
	UZH3	29.6	0.03378
	UZH4	16.38	0.06105
	UZH5	21.98	0.04550
	UZH6	32.63	0.03065
	UZH7	18.49	0.05408
	UZH8	23.11	0.04327
	UZH9	21.75	0.04598
	UZH10	17.44	0.05734
	UZH11	28.17	0.03550
	UZH12	19.97	0.05008
	Average	24.8725	0.04021
	Shapiro-Wilk normality test	W = 0.93295	$P = 0.4124$
2[b]	Li-1	26.165	0.03822
	Li-2	29.958	0.03338
	Chen-3	36.397	0.02747
	Chen-4	Missing	
	Average	30.84	0.03306
	Shapiro-Wilk normality test	W = 0.99671	$P = 0.8904$

[a] E gene was the target for this sample set; [b] F: N gene was the target for this sample set.

Table S2. Primers list of this study

Target gene		Institute	Primer name	Type	Position	Primer sequence（5’-3’）	Size (bp)	T_m (°C) [a]
E		Charité	E_Sarbeco	F[b]	26269-26294	ACAGGTACGTTAATAGTTAATAGCGT	125	58
				R[c]	26360-26381	ATATTGCAGCAGTACGCACACA		
				P[d]	26332-26357	Fam-ACACTAGCCATCCTTACTGCGCTTCG-BHQ-1 [e]		
ORF1	*RdRp*	IP	nCoV_IP2	F[b]	12669-12687	ATGAGCTTAGTCCTGTTG	108	
				R[c]	12759-12777	CTCCCTTTGTTGTGTTGT		
				P[d]	12696-12717	FAM-AGATGTCTTGTGCTGCCGGTA-BHQ1 [e]		
			nCoV_IP4	F[b]	14059-14078	GGTAACTGGTATGATTTCG	107	
				R[c]	14146-14166	CTGGTCAAGGTTAATATAGG		
				P[d]	14084-14103	FAM-TCATACAAACCACGCCAGG-BHQ1 [e]		
		Charité	RdRp-SARS	F[b]	15431-15452	GTGARATGGTCATGTGTGGCGG	100	
				R[c]	15505-15530	CARATGTTAAASACACTATTAGCATA		
				P[d]	15470-15594	FAM-CCAGGTGGWACRTCATCMGGTGATGC-BHQ1 [e]		
			RdRp-CoV-19	F[b]	15431-15452	GTGARATGGTCATGTGTGGCGG		
				R[c]	15505-15530	CARATGTTAAAAACACTATTAGCATA		
				P[d]	15471-15594	FAM-CAGGTGGAACCTCATCAGGAGATGC-BHQ1 [e]		
		China CDC	ORF1ab	F[b]	13342-13362	CCCTGTGGGTTTTACACTTAA	119	55
				R[c]	13442-13460	ACGATTGTGCATCAGCTGA		
				P[d]	13377-13404	FAM-CCGTCTGCGGTATGTGGAAAGGTTATGG-BHQ1 [e]		
	nsp14	HKU	ORF1b-nsp14	F[b]	18778-18797	TGGGGYTTTACRGGTAACCT	130	60
				R[c]	18889-18909	AACRCGCTTAACAAAGCACTC		
				P[d]	18849-18872	FAM-TAGTTGTGATGCWATCATGACTAG-BHQ1 [e]		
N			HKU-N	F[b]	29145-29166	TAATCAGACAAGGAACTGATTA	110	
				R[c]	29235-29254	CGAAGGTGTGACTTCCATG		
				P[d]	29177-29196	FAM-GCAAATTGTGCAATTTGCGG-BHQ1 [e]		

Japan NIID	NIID_2019-nCoV_2	F[b]	29125-29144	AAATTTTGGGGACCAGGAAC		
		R[c]	29263-29282	TGGCAGCTGTGTAGGTCAAC [f]	158	
		P[d]	29222-29241	FAM-ATGTCGCGCATTGGCATGGA-BHQ1 [e]		
China CDC	N	F[b]	28881-28902	GGGGAACTTCTCCTGCTAGAAT		
		R[c]	28958-28979	CAGACATTTTGCTCTCAAGCTG	99	
		P[d]	28934-28953	FAM-TTGCTGCTGCTTGACAGATT-BHQ1 [e]		
US CDC	2019-nCoV_N1	F[b]	28287-28306	GACCCCAAAATCAGCGAAAT		
		R[c]	28335-28358	TCTGGTTACTGCCAGTTGAATCTG	72	
		P[d]	28309-28332	FAM-ACCCCGCATTACGTTTGGTGGACC-BHQ1 [e]		
	2019-nCoV_N2	F[b]	29164-29183	TTACAAACATTGGCCGCAAA		
		R[c]	29213-29230	GCGCGACATTCCGAAGAA	66	55
		P[d]	29188-29210	FAM-ACAATTTGCCCCCAGCGCTTCAG-BHQ1 [e]		
	2019-nCoV_N3	F[b]	28681-28702	GGGAGCCTTGAATACACCAAA A		
		R[c]	28732-28752	TGTAGCACGATTGCAGCATTG	73	
		P[d]	28704-28727	FAM-AYCACATTGGCACCCGCAATCCTG-BHQ1 [e]		
Thailand NIH	WH-NIC N	F[b]	28320-28339	CGTTTGGTGGACCCTCAGAT		
		R[c]	28358-28376	CCCCACTGCGTTCTCCATT	57	
		P[d]	28341-28356	FAM-CAACTGGCAGTAACCA-BQH1 [e]		

[a] T_m: melting temperature for PCR and SYBR Green-based qPCR, same as the extension temperature for TaqMan-based RT-qPCR; [b] F: forward primer; [c] R: reverse primer; [d] P: probe; [e] FAM: 6-carboxyfluorescein; BHQ-1: Black Hole Quencher-1; [f] The underlined letter within NIID_2019-nCoV_NR2 indicates a mismatched site to the PCR template, which should be C .

Table S3. The *P* values of Shapiro-Wilk test on the reciprocals of the Ct values of undiluted and diluted samples with different methods

Primer name	Undiluted samples		Diluted samples		Both samples	
	TaqMan	SYBR Green	TaqMan	SYBR Green	TaqMan	SYBR Green
ORF1ab	4.27E-06*	1.25E-02*	3.11E-05*	2.89E-05*	1.985E-08*	9.292E-08*
N	7.45E-05*	2.26E-07*	6.53E-08*	2.21E-07*	1.479E-09*	6.82E-12*
nCoV_IP4	3.47E-02*	1.63E-07*	7.61E-07*	3.68E-03*	4.201E-06*	1.325E-11*
2019-nCoV_N1	4.13E-05*	1.89E-03*	2.29E-07*	4.83E-02*	5.268E-09*	2.437E-08*
2019-nCoV_N3	1.16E-04*	7.52E-03*	2.29E-05*	6.01E-04*	2.694E-08*	4.028E-06*
NIID_2019-nCoV_2	1.79E-04*	2.12E-07*	6.26E-07*	1.25E-05*	4.95E-08*	2.513E-11*
ORF1b-nsp14	8.79E-06*	1.72E-08*	6.80E-06*	3.25E-02*	1.045E-08*	2.278E-13*
HKU-N	5.16E-05*	5.58E-08*	4.78E-01	6.58E-01	2.959E-10*	5.87E-13*
WH-NIC N	2.02E-07*	1.07E-06*	8.40E-09*	3.84E-03*	1.087E-11*	3.426E-09*

*: significant ($P<0.05$)

Table S4. The averages and *P* values of Randomization test or Paired Student's *t* test on the reciprocals of the Ct values of undiluted and diluted samples matched with different methods

Primer name	Undiluted samples			Diluted samples			Both samples		
	Average		*P* value	Average		*P* value	Average		*P* value
	TaqMan	SYBR Green		TaqMan	SYBR Green		TaqMan	SYBR Green	
ORF1ab	0.0089	0.031	0.00E+00[a, *]	0.013	0.033	0.00E+00[a, *]	0.011	0.032	0.00E+00[a, *]
N	0.031	0.033	3.22E-01[a]	0.030	0.037	1.00E-04[a, *]	0.030	0.035	0.0071 [a, *]
nCoV_IP4	0.030	0.029	5.29E-01[a]	0.030	0.033	0.00E+00[a, *]	0.030	0.031	0.4214 [a]
2019-nCoV_N1	0.028	0.035	0.00E+00[a, *]	0.028	0.035	0.00E+00[a, *]	0.028	0.035	0.00E+00 [a, *]
2019-nCoV_N3	0.029	0.034	1.15E-02[a, *]	0.029	0.032	9.00E-04[a, *]	0.029	0.033	1.00E-04 [a, *]
NIID_2019-nCoV_2	0.017	0.033	4.00E-04[a, *]	0.0072	0.037	0.00E+00[a, *]	0.012	0.035	0.00E+00[a, *]
ORF1b-nsp14	0.017	0.043	0.00E+00[a, *]	0.018	0.047	0.00E+00[a, *]	0.017	0.045	0.00E+00[a, *]
HKU-N	0.026	0.043	0.00E+00[a, *]	0.044	0.046	6.55E-08[b, *]	0.035	0.044	0.00E+00[a, *]

WH-NIC N	0.031	0.039	7.50E-03[a, *]	0.032	0.040	0.00E+00[a, *]	0.031	0.039	0.00E+00[a, *]

[a]: Randomization test; [b]: Paired Student's t test; [*]: significant ($P<0.05$)

Table S5. The P values of post-hoc analysis of Friedman test on the reciprocals of the Ct values of undiluted samples measured with different primer sets by SYBR Green method (Friedman chi-squared = 117.84, df = 8, P < 2.2E-16[a])

	2019-nCoV_N1	2019-nCoV_N3	HKU-N	N	nCoV_IP4	NIID_2019-nCoV_2	ORF1ab	ORF1b-nsp14
2019-nCoV_N3	0.98859	-	-	-	-	-	-	-
HKU-N	0.00516[*]	7.50E-05[*]	-	-	-	-	-	-
N	0.98089	0.52994	0.14104	-	-	-	-	-
nCoV_IP4	0.10031	0.62468	1.30E-09[*]	0.00306[*]	-	-	-	-
NIID_2019-nCoV_2	0.96508	1	3.00E-05[*]	0.40067	0.74934	-	-	-
ORF1ab	0.01662[*]	0.24333	2.00E-11[*]	0.00024[*]	0.99965	0.34921	-	-
ORF1b-nsp14	0.00246[*]	3.00E-05[*]	1	0.08689	3.50E-10[*]	1.10E-05[*]	4.90E-12[*]	-
WH-NIC N	0.20491	0.01256[*]	0.9543	0.85251	2.20E-06[*]	0.00633[*]	7.00E-08[*]	0.89833

[a]: the result of Friedman test; [*]: significant ($P<0.05$)

Table S6. The P values of post-hoc analysis of Friedman test on the reciprocals of the Ct values of undiluted samples measured with different primer sets by TaqMan method (Friedman chi-squared = 99.002, df = 8, P < 2.2E-16[a])

	2019-nCoV_N1	2019-nCoV_N3	HKU-N	N	nCoV_IP4	NIID_2019-nCoV_2	ORF1ab	ORF1b-nsp14
2019-nCoV_N3	1	-	-	-	-	-	-	-
HKU-N	0.99983	0.99584	-	-	-	-	-	-
N	0.88783	0.73256	0.99362	-	-	-	-	-
nCoV_IP4	0.99999	1	0.99219	0.67993	-	-	-	-
NIID_2019-nCoV_2	0.0138[*]	0.0367[*]	0.00177[*]	3.00E-05[*]	0.04697[*]	-	-	-
ORF1ab	0.00014[*]	0.00056[*]	9.70E-06[*]	5.90E-08[*]	0.0008[*]	0.97387	-	-
ORF1b-nsp14	0.00221[*]	0.007[*]	0.00021[*]	2.20E-06[*]	0.00941[*]	0.99992	0.99951	-
WH-NIC N	0.49214	0.30119	0.83948	0.99933	0.2571	9.00E-07[*]	8.80E-10[*]	5.00E-08[*]

[a]: the result of Friedman test; [*]: significant ($P<0.05$)

Table S7. The *P* values of Shapiro-Wilk test on the reciprocals of the Ct values of positive and negative undiluted samples with different methods

**Primer name**	**Positive samples**		**Negative samples**	
	**TaqMan**	**SYBR Green**	**TaqMan**	**SYBR Green**
ORF1ab	2.44E-03*	6.02E-01	1.05E-06*	1.12E-03*
N	2.73E-02*	2.40E-03*	3.68E-05*	5.32E-05*
nCoV_IP4	6.11E-03*	1.44E-05*	1.65E-02*	7.90E-06*
2019-nCoV_N1	2.69E-01	2.74E-03*	5.33E-04*	1.17E-03*
2019-nCoV_N3	2.61E-02*	9.73E-03*	3.82E-05*	8.79E-02
NIID_2019-nCoV_2	3.86E-03	3.07E-01	1.70E-04*	1.95E-04*
ORF1b-nsp14	1.78E-05*	1.12E-01	2.68E-04*	3.38E-06*
HKU-N	4.51E-02*	1.19E-05*	4.11E-05*	3.97E-05*
WH-NIC N	1.06E-04*	6.09E-02	3.00E-07*	1.54E-05

*: significant (P<0.05)

Table S8. The averages and *P* values of Randomization test on the reciprocals of the Ct values of positive and negative undiluted samples measured by different methods

**Primer name**	**TaqMan**			**SYBR Green**		
	**Average**		**P value**	**Average**		**P value**
	**Positive**	**Negative**		**Positive**	**Negative**	
ORF1ab	0.013	0.0046	1.39E-01	0.032	0.031	9.90E-02
N	0.034	0.027	4.00E-04*	0.038	0.025	4.00E-04*
nCoV_IP4	0.031	0.029	5.80E-02	0.031	0.026	2.16E-01
2019-nCoV_N1	0.033	0.023	4.00E-03*	0.037	0.034	4.00E-04*
2019-nCoV_N3	0.035	0.023	0.00E+00*	0.035	0.033	1.48E-02*
NIID_2019-nCoV_2	0.024	0.0091	8.80E-03*	0.035	0.030	7.06E-02
ORF1b-nsp14	0.023	0.010	4.40E-03*	0.047	0.039	0.00E+00*
HKU-N	0.043	0.0076	0.00E+00*	0.046	0.039	1.01E-01
WH-NIC N	0.032	0.030	5.00E-01	0.040	0.038	7.38E-01

*: significant (P<0.05)

Table S9. The *P* values of post-hoc analysis of Friedman test on the reciprocals of the Ct values of positive undiluted samples measured with different primer sets and by different methods (Friedman chi-squared = 178.24, df = 18, *P*< 2.2E-16[a])

**Primer name**	**Primer name**	**2019-nCoV_N1**		**2019-nCoV_N3**		**HKU-N**		**Hosptal result**	**N**		**nCoV_IP4**		**NIID_2019-nCoV_2**		**ORF1ab**		**ORF1b-nsp14**		**WH-NIC N**
	**Method**	**SYBR**	**TaqMan**	**SYBR**	**TaqMan**	**SYBR**	**TaqMan**	**TaqMan**	**SYBR**	**TaqMan**	**SYBR**	**TaqMan**	**SYBR**	**TaqMan**	**SYBR**	**TaqMan**	**SYBR**	**TaqMan**	**SYBR**
2019-nCoV_N1	TaqMan	0.61213	-	-	-	-	-	-	-	-	-	-	-	-	-	-	-	-	-
2019-nCoV_N3	SYBR	1	0.98653	-	-	-	-	-	-	-	-	-	-	-	-	-	-	-	-
	TaqMan	0.66765	1	0.99197	-	-	-	-	-	-	-	-	-	-	-	-	-	-	-
HKU-N	SYBR	0.93077	0.00238*	0.38947	0.00325*	-	-	-	-	-	-	-	-	-	-	-	-	-	-
	TaqMan	0.99915	0.02693*	0.83615	0.03463*	1	-	-	-	-	-	-	-	-	-	-	-	-	-
Hosptal Result	TaqMan	1	0.52696	0.99999	0.58385	0.95966	0.99975	-	-	-	-	-	-	-	-	-	-	-	-
N	SYBR	1	0.26983	0.99877	0.31488	0.99665	1	1	-	-	-	-	-	-	-	-	-	-	-
	TaqMan	0.99826	0.9999	1	0.99996	0.1195	0.47062	0.99545	0.95127	-	-	-	-	-	-	-	-	-	-
nCoV_IP4	SYBR	0.32676	1	0.89756	1	0.00042*	0.00638*	0.25921	0.10246	0.99473	-	-	-	-	-	-	-	-	-
	TaqMan	0.05593	0.99994	0.443	0.99984	1.30E-05*	0.00032*	0.03915*	0.01054*	0.81549	1	-	-	-	-	-	-	-	-
NIID_2019-nCoV_2	SYBR	0.99942	0.99961	1	0.99984	0.16012	0.5554	0.99826	0.97316	1	0.98811	0.74605	-	-	-	-	-	-	-
	TaqMan	0.00203*	0.91853	0.04976*	0.88985	7.50E-08*	3.20E-06*	0.00125*	0.00023*	0.21011	0.99082	1	0.16012	-	-	-	-	-	-
	SYB	0.1195	1	0.64012	1	5.20E-05*	0.00106*	0.08742	0.02693*	0.93077	1	1	0.88985	0.99984	-	-	-	-	-

	R																		
ORF1ab	TaqMan	5.20E-05*	0.41592	0.00278*	0.36376	4.30E-10*	2.90E-08*	2.90E-05*	3.90E-06*	0.02079*	0.70777	0.98286	0.0139*	1	0.93077	-	-	-	-
ORF1b-nsp14	SYBR	0.83615	0.0009*	0.24885	0.00125*	1	1	0.88985	0.98286	0.06273	0.00014*	3.90E-06*	0.08742	1.80E-08*	1.60E-05*	9.00E-11*	-	-	-
	TaqMan	0.00032*	0.69457	0.01212*	0.64012	5.40E-09*	2.90E-07*	0.00019*	2.90E-05*	0.07021	0.91189	0.99915	0.04976*	1	0.99197	1	1.20E-09*	-	-
WH-NIC N	SYBR	0.99999	0.08742	0.96693	0.10791	0.99998	1	1	1	0.74605	0.02526*	0.00173*	0.81549	2.40E-05*	0.00511*	2.90E-07*	0.99961	2.60E-06*	-
	TaqMan	0.97591	1	1	1	0.0416*	0.23876	0.95561	0.80466	1	0.99984	0.95561	1	0.42939	0.99082	0.06638	0.01946*	0.18391	0.48459

[a]: the result of Friedman test; *: significant ($P<0.05$)

Table S10. The P values of post-hoc analysis of Friedman test on the reciprocals of the Ct values of diluted samples measured with different primer sets by SYBR Green method (Friedman chi-squared = 151.78, df = 8, $P < 2.2E\text{-}16^{a}$)

	**2019-nCoV_N1**	**2019-nCoV_N3**	**HKU-N**	**N**	**nCoV_IP4**	**NIID_2019-nCoV_2**	**ORF1ab**	**ORF1b-nsp14**
2019-nCoV_N3	0.39659	-	-	-	-	-	-	-
HKU-N	2.00E-06*	8.20E-13*	-	-	-	-	-	-
N	0.46755	0.00053*	0.02527*	-	-	-	-	-
nCoV_IP4	0.92643	0.99324	5.50E-10*	0.01779*	-	-	-	-
NIID_2019-nCoV_2	0.61559	0.00131*	0.01235*	1	0.03539*	-	-	-
ORF1ab	0.90909	0.99553	3.80E-10*	0.01485*	1	0.02996*	-	-
ORF1b-nsp14	3.20E-09*	7.40E-14*	0.98039	0.00041*	2.70E-13*	0.00016*	2.80E-13*	-
WH-NIC N	0.03539*	2.00E-06*	0.39659	0.97341	0.0002*	0.92643	0.00016*	0.02996*

[a]: the result of Friedman test; *: significant ($P<0.05$)

Table S11. The P values of post-hoc analysis of Friedman test on the reciprocals of the Ct values of diluted samples measured with different primer sets by TaqMan method (Friedman chi-squared = 150.98, df = 8, $P < 2.2E\text{-}16^{a}$)

	**2019-nCoV_N1**	**2019-nCoV_N3**	**HKU-N**	**N**	**nCoV_IP4**	**NIID_2019-nCoV_2**	**ORF1ab**	**ORF1b-nsp14**
2019-nCoV_N3	0.99324	-	-	-	-	-	-	-

HKU-N	0.00012*	1.10E-06*	-	-	-	-	-	-
N	0.48582	0.07154	0.20542	-	-	-	-	-
nCoV_IP4	1	0.99009	0.00016*	0.52276	-	-	-	-
NIID_2019-nCoV_2	0.00083*	0.02527*	6.60E-14*	1.10E-08*	0.00066*	-	-	-
ORF1ab	0.00309*	0.06641	8.30E-14*	7.90E-08*	0.00251*	1	-	-
ORF1b-nsp14	0.01235*	0.17197	1.20E-13*	7.10E-07*	0.01023*	0.99897	0.99999	-
WH-NIC N	0.55996	0.0954	0.16173	1	0.59713	2.10E-08*	1.50E-07*	1.30E-06*

[a]: the result of Friedman test; *: significant (P<0.05)

Table S12. Mismatches in the primer and probe targets of diagnostic PCR assays with Delta variant

Target gene	Institute	Primer name	Type	Group number	Variant count	Frequency (%)	Sequence (5'-3')
E	Charité	E_Sarbeco	F	Total sequences	10106		ACAGGTACGTTAATAGTTAATAGCGT
				Informative sequences	10093		10 20
				1	10076	99.703	....|....|....|....|....|.
				2	10	0.099	..........g...............
				3	3	0.030	.................c........
				4	3	0.030	.t........................
				5	1	0.010	........a.................
				Outgroup1	13	0.129	
			R	Total sequences	10106		TGTGTGCGTACTGCTGCAATAT
				Informative sequences	10068		10 20
				1	10065	99.594	....|....|....|....|..
				2	2	0.020	..........t...........
				3	1	0.010	....................g.
				Outgroup1	38	0.376	
			P	Total sequences	10106		ACACTAGCCATCCTTACTGCGCTTCG
				Informative sequences	10085		10 20
				1	10083	99.772	....|....|....|....|....|.
				2	1	0.010	........t.................

					3	1	0.010	...................t......
					Outgroup1	21	0.208	
Orf1	RdRp	IP	nCoV_IP2	F	Total sequences	10106		ATGAGCTTAGTCCTGTTG
					Informative sequences	10104		10
					1	10104	99.980	....|....|....|...
					Outgroup1	2	0.020	
				R	Total sequences	10106		ACAACACAACAAAGGGAG
					Informative sequences	10057		10
					1	10021	99.159	....|....|....|...
					2	25	0.247	....t.............
					3	8	0.079	.........t........
					4	2	0.020	.t................
					5	1	0.010	.................a
					Outgroup1	49	0.485	
				P	Total sequences	10106		AGATGTCTTGTGCTGCCGGTA
					Informative sequences	10102		10 20
					1	10068	99.624	....|....|....|....|.
					2	33	0.327	.t...................
					3	1	0.010	...c.................
					Outgroup1	4	0.040	
			nCoV_IP4	F	Total sequences	10106		GGTAACTGGTATGATTTCG
					Informative sequences	10092		10
					1	10092	99.861	....|....|....|....
					Outgroup1	14	0.139	
				R	Total sequences	10106		CCTATATTAACCTTGACCAG
					Informative sequences	10090		10 20
					1	9762	96.596	....|....|....|....|
					2	162	1.603	...g................
					3	152	1.504	...................t
					4	5	0.049	................t...
					5	4	0.040	.................t..
					6	3	0.030	...........t........
					7	1	0.010	..............t.....
					8	1	0.010	..........t.........

			Outgroup1	16	0.158	...g................
		P	Total sequences	10106		TCATACAAACCACGCCAGG
			Informative sequences	10090		10
			1	10030	99.248	....|....|....|....
			2	35	0.346	.................a.
			3	19	0.188	...............t...
			4	2	0.020	.............a.....
			5	2	0.020	..........a........
			6	1	0.010	..................t
			7	1	0.010	..g................
			Outgroup1	16	0.158	
Charité	RdRp-SARS & RdRp-CoV-19	F	Total sequences	10106	RdRp-SARS<br>RdRp-CoV-19	GTGARATGGTCATGTGTGGCGG<br>GTGAAATGGTCATGTGTGGCGG
			Informative sequences	10078		10 20
			1	9598	94.973	....|....|....|....|a.
			2	474	4.690	....
			3	4	0.040	.................t....
			4	2	0.020	.......t..............
			Outgroup1	28	0.277	
		R	Total sequences	10106	RdRp-SARS<br>RdRp-CoV-19	TATGCTAATAGTGTSTTTAACATRTG<br>TATGCTAATAGTGTTTTTAACATRTG
			Informative sequences	10102		10 20
			1	10102	99.960	....|....|....|....|...t|.
			Outgroup1	4	0.040	
		P	Total sequences	10106	RdRp-SARS<br>RdRp-CoV-19	CCAGGTGGAACCTCATCAGGAGATGC<br>CAGGTGGAACCTCATCAGGAGATGC
			Informative sequences	10082		10 20
			1	10081	99.753	....|....|....|....|....|.
			2	1	0.010	..g.......................
			Outgroup1	24	0.237	
China CDC	ORF1ab	F	Total sequences	10106		CCCTGTGGGTTTTACACTTAA

				Informative sequences	10096		`         10        20`
				1	10094	99.881	`....|....|....|....|.`
				2	2	0.020	`.....t...............`
				Outgroup1	10	0.099	
			R	Total sequences	10106		TCAGCTGATGCACAATCGT
				Informative sequences	10038		`         10`
				1	10021	99.159	`....|....|....|....`
				2	13	0.129	`................t..`
				3	2	0.020	`.................t.`
				4	1	0.010	`.....c.............`
				5	1	0.010	`...............c...`
				Outgroup1	68	0.673	
			P	Total sequences	10106		CCGTCTGCGGTATGTGGAAAGGTTATGG
				Informative sequences	10058		`         10        20`
				1	10056	99.505	`....|....|....|....|....|...`
				2	1	0.010	`..........................t.`
				3	1	0.010	`..t.........................`
				Outgroup1	48	0.475	
nsp14	HKU	ORF1b-nsp14	F	Total sequences	10106		TGGGGTTTTACAGGTAACCT
				Informative sequences	10103		`         10        20`
				1	10097	99.911	`....|....|....|....|`
				2	2	0.020	`...t................`
				3	2	0.020	`..........t.........`
				4	1	0.010	`....t...............`
				5	1	0.010	`...........g........`
				Outgroup1	3	0.030	
			R	Total sequences	10106		GAGTGCTTTGTTAAGCGRGTT
				Informative sequences	9933		`         10        20`
				1	9919	98.150	`....|....|....|..t.|.`
				2	5	0.049	`...........c.....t...`
				3	5	0.049	`.................tt..`
				4	3	0.030	`.........t.......t...`
				5	1	0.010	`..t..............t...`
				Outgroup1	170	1.682	

<table>
<tr><td></td><td></td><td></td><td>Outgroup2</td><td>3</td><td>0.030</td><td></td></tr>
<tr><td></td><td></td><td>P</td><td>Total sequences</td><td>10106</td><td></td><td>TAGTTGTGATGCAATCATGACTAG</td></tr>
<tr><td></td><td></td><td></td><td>Informative sequences</td><td>9936</td><td></td><td>10 20</td></tr>
<tr><td></td><td></td><td></td><td>1</td><td>9935</td><td>98.308</td><td>....|....|....|....|....</td></tr>
<tr><td></td><td></td><td></td><td>2</td><td>1</td><td>0.010</td><td>.............c..........</td></tr>
<tr><td></td><td></td><td></td><td>Outgroup1</td><td>170</td><td>1.682</td><td></td></tr>
<tr><td>N</td><td>HKU-N</td><td>F</td><td>Total sequences</td><td>10106</td><td></td><td>TAATCAGACAAGGAACTGATTA</td></tr>
<tr><td></td><td></td><td></td><td>Informative sequences</td><td>10095</td><td></td><td>10 20</td></tr>
<tr><td></td><td></td><td></td><td>1</td><td>10087</td><td>99.812</td><td>....|....|....|....|..</td></tr>
<tr><td></td><td></td><td></td><td>2</td><td>3</td><td>0.030</td><td>....t.................</td></tr>
<tr><td></td><td></td><td></td><td>3</td><td>2</td><td>0.020</td><td>....................c.</td></tr>
<tr><td></td><td></td><td></td><td>4</td><td>2</td><td>0.020</td><td>....a.................</td></tr>
<tr><td></td><td></td><td></td><td>5</td><td>1</td><td>0.010</td><td>...................c..</td></tr>
<tr><td></td><td></td><td></td><td>Outgroup1</td><td>11</td><td>0.109</td><td></td></tr>
<tr><td></td><td></td><td>R</td><td>Total sequences</td><td>10106</td><td></td><td>CATGGAAGTCACACCTTCG</td></tr>
<tr><td></td><td></td><td></td><td>Informative sequences</td><td>10090</td><td></td><td>10</td></tr>
<tr><td></td><td></td><td></td><td>1</td><td>9983</td><td>98.783</td><td>....|....|....|....</td></tr>
<tr><td></td><td></td><td></td><td>2</td><td>59</td><td>0.584</td><td>.................t.</td></tr>
<tr><td></td><td></td><td></td><td>3</td><td>24</td><td>0.237</td><td>..................t</td></tr>
<tr><td></td><td></td><td></td><td>4</td><td>11</td><td>0.109</td><td>.............t.....</td></tr>
<tr><td></td><td></td><td></td><td>5</td><td>8</td><td>0.079</td><td>...a...............</td></tr>
<tr><td></td><td></td><td></td><td>6</td><td>2</td><td>0.02</td><td>...........t.......</td></tr>
<tr><td></td><td></td><td></td><td>7</td><td>1</td><td>0.01</td><td>...t...............</td></tr>
<tr><td></td><td></td><td></td><td>8</td><td>1</td><td>0.01</td><td>..................a</td></tr>
<tr><td></td><td></td><td></td><td>9</td><td>1</td><td>0.01</td><td>..............t....</td></tr>
<tr><td></td><td></td><td></td><td>Outgroup1</td><td>16</td><td>0.158</td><td></td></tr>
<tr><td></td><td></td><td>P</td><td>Total sequences</td><td>10106</td><td></td><td>CCGCAAATTGCACAATTTGC</td></tr>
<tr><td></td><td></td><td></td><td>Informative sequences</td><td>10091</td><td></td><td>10 20</td></tr>
<tr><td></td><td></td><td></td><td>1</td><td>10086</td><td>99.802</td><td>....|....|....|....|</td></tr>
<tr><td></td><td></td><td></td><td>2</td><td>3</td><td>0.030</td><td>..t.................</td></tr>
<tr><td></td><td></td><td></td><td>3</td><td>1</td><td>0.010</td><td>..............g.....</td></tr>
<tr><td></td><td></td><td></td><td>4</td><td>1</td><td>0.010</td><td>..c.................</td></tr>
<tr><td></td><td></td><td></td><td>Outgroup1</td><td>15</td><td>0.148</td><td></td></tr>
<tr><td>Japan NIID</td><td>NIID_2019-</td><td>F</td><td>Total sequences</td><td>10106</td><td></td><td>AAATTTTGGGGACCAGGAAC</td></tr>
</table>

	nCoV_2		Informative sequences	10094		10 20
			1	10086	99.802	....|....|....|....|
			2	4	0.040	...............t....
			3	3	0.030	...................t
			4	1	0.010	.........a..........
			Outgroup1	12	0.119	
		R	Total sequences	10106		GTTGACCTACACAGCTGCCA[a]
			Informative sequences	10084		10 20
			1	10061	99.555	....|....|....g....|
			2	5	0.049	......t.......g.....
			3	4	0.040	.....t........g.....
			4	4	0.040	.........t....g.....
			5	4	0.040	...a..........g.....
			6	2	0.020	...........t..g.....
			7	2	0.020	...c..........g.....
			8	1	0.010	.c............g.....
			9	1	0.010	..............g....g
			Outgroup1	22	0.218	
		P	Total sequences	10106		ATGTCGCGCATTGGCATGGA
			Informative sequences	10088		10 20
			1	10060	99.545	....|....|....|....|
			2	19	0.188	.....t..............
			3	8	0.079	.................a..
			4	1	0.010	.................t..
			Outgroup1	18	0.178	
China CDC	N	F	Total sequences	10106		GGGGAACTTCTCCTGCTAGAAT
			Informative sequences	10004		10 20
			1	9932	98.278	t...|....|....|....|..
			2	24	0.237	....
			3	9	0.089	aac...................
			4	7	0.069	t.................t...
			5	7	0.069	t...............c.....
			6	3	0.030	t....................c

	7	3	0.030	t.............t.......
	8	2	0.020	t.a...................
	9	2	0.020	t........t............
	10	2	0.020	c.....................
	11	2	0.020	t.....t...............
	12	2	0.020	t..........t..........
	13	2	0.020	t..............t......
	14	1	0.010	t...................g.
	15	1	0.010	t.................a...
	16	1	0.010	t.............a.......
	17	1	0.010	t.................c...
	18	1	0.010	t..t..................
	19	1	0.010	ta....................
	20	1	0.010	......t...............
	Outgroup1	101	0.999	
	Outgroup2	1	0.010	
	Total sequences	10106		CAGCTTGAGAGCAAAATGTCTG
	Informative sequences	10008		10 20
	1	9950	98.456	....|....|....|....|..
	2	27	0.267	...................t..
	3	16	0.158	....................c.
	4	3	0.030	..t...................
	5	2	0.020	.................t....
R	6	2	0.020	.....................a
	7	2	0.020	...t..................
	8	1	0.010	........t.............
	9	1	0.010	.............g........
	10	1	0.010	........a.............
	11	1	0.010	.....a................
	12	1	0.010	.................a....
	13	1	0.010	...............g......
	Outgroup1	98	0.970	
P	Total sequences	10106		TTGCTGCTGCTTGACAGATT

			Informative sequences	10017		10 20
			1	9994	98.892	....|....|....|....|
			2	18	0.178	.....a..............
			3	2	0.020	.................g..
			4	1	0.010	.ca.c...............
			5	1	0.010	..............t.....
			6	1	0.010	......a.............
			Outgroup1	89	0.881	
US CDC	2019-nCoV_N1	F	Total sequences	10106		GACCCCAAAATCAGCGAAAT
			Informative sequences	10084		10 20
			1	10059	99.535	....|....|....|....|
			2	7	0.069	.............t......
			3	7	0.069	....t...............
			4	3	0.030	..........c.........
			5	2	0.020	...t................
			6	1	0.010	..t.................
			7	1	0.010	...............c....
			8	1	0.010	.....a..............
			9	1	0.010	.......t............
			10	1	0.010	.............a......
			11	1	0.010	...............t....
			Outgroup1	22	0.218	
		R	Total sequences	10106		CAGATTCAACTGGCAGTAACCAGA
			Informative sequences	10073		10 20
			1	10064	99.584	....|....|....|....|....
			2	3	0.030	.........t..............
			3	2	0.020	...............t........
			4	2	0.020	...................a....
			5	1	0.010	...................t....
			6	1	0.010	..................g.....
			Outgroup1	33	0.327	
		P	Total sequences	10106		ACCCCGCATTACGTTTGGTGGACC
			Informative sequences	10073		10 20

<table>
<tr><th></th><th></th><th></th><th></th><th></th><th></th></tr>
<tr><td></td><td></td><td>1</td><td>9946</td><td>98.417</td><td>....|....|....|....|....</td></tr>
<tr><td></td><td></td><td>2</td><td>72</td><td>0.712</td><td>................t.......</td></tr>
<tr><td></td><td></td><td>3</td><td>18</td><td>0.178</td><td>.a......................</td></tr>
<tr><td></td><td></td><td>4</td><td>14</td><td>0.139</td><td>.................t......</td></tr>
<tr><td></td><td></td><td>5</td><td>8</td><td>0.079</td><td>......t.................</td></tr>
<tr><td></td><td></td><td>6</td><td>3</td><td>0.030</td><td>....t...................</td></tr>
<tr><td></td><td></td><td>7</td><td>2</td><td>0.020</td><td>..t.....................</td></tr>
<tr><td></td><td></td><td>8</td><td>2</td><td>0.020</td><td>...........a............</td></tr>
<tr><td></td><td></td><td>9</td><td>2</td><td>0.020</td><td>...t....................</td></tr>
<tr><td></td><td></td><td>10</td><td>2</td><td>0.020</td><td>.......................t</td></tr>
<tr><td></td><td></td><td>11</td><td>1</td><td>0.010</td><td>.....a..................</td></tr>
<tr><td></td><td></td><td>12</td><td>1</td><td>0.010</td><td>...........t............</td></tr>
<tr><td></td><td></td><td>13</td><td>1</td><td>0.010</td><td>..a.....................</td></tr>
<tr><td></td><td></td><td>14</td><td>1</td><td>0.010</td><td>....................t...</td></tr>
<tr><td></td><td></td><td>Outgroup1</td><td>33</td><td>0.327</td><td></td></tr>
<tr><td rowspan="21">2019-nCoV_N2</td><td rowspan="9">F</td><td>Total sequences</td><td>10106</td><td></td><td>TTACAAACATTGGCCGCAAA</td></tr>
<tr><td>Informative sequences</td><td>10090</td><td></td><td>10 20</td></tr>
<tr><td>1</td><td>10071</td><td>99.654</td><td>....|....|....|....|</td></tr>
<tr><td>2</td><td>12</td><td>0.119</td><td>...t................</td></tr>
<tr><td>3</td><td>3</td><td>0.030</td><td>...............t....</td></tr>
<tr><td>4</td><td>2</td><td>0.020</td><td>.c..................</td></tr>
<tr><td>5</td><td>1</td><td>0.010</td><td>...............c....</td></tr>
<tr><td>6</td><td>1</td><td>0.010</td><td>c...................</td></tr>
<tr><td>Outgroup1</td><td>16</td><td>0.158</td><td></td></tr>
<tr><td rowspan="6">R</td><td>Total sequences</td><td>10106</td><td></td><td>TTCTTCGGAATGTCGCGC</td></tr>
<tr><td>Informative sequences</td><td>10089</td><td></td><td>10</td></tr>
<tr><td>1</td><td>10055</td><td>99.495</td><td>....|....|....|...</td></tr>
<tr><td>2</td><td>19</td><td>0.188</td><td>..............t...</td></tr>
<tr><td>3</td><td>15</td><td>0.148</td><td>..t...............</td></tr>
<tr><td>Outgroup1</td><td>17</td><td>0.168</td><td></td></tr>
<tr><td rowspan="3">P</td><td>Total sequences</td><td>10106</td><td></td><td>ACAATTTGCCCCCAGCGCTTCAG</td></tr>
<tr><td>Informative sequences</td><td>10089</td><td></td><td>10 20</td></tr>
<tr><td>1</td><td>10081</td><td>99.753</td><td>....|....|....|....|...</td></tr>
</table>

		2	3	0.030	............t..........
		3	1	0.010	...g...................
		4	1	0.010	................t......
		5	1	0.010	...............t.......
		6	1	0.010	.............g.........
		7	1	0.010	..............a........
		Outgroup1	17	0.168	
2019-nCoV_N3	F	Total sequences	10106		GGGAGCCTTGAATACACCAAAA
		Informative sequences	10083		10 20
		1	10017	99.119	....|....|....|....|..
		2	45	0.445	.........t............
		3	5	0.049	............c.........
		4	4	0.040	t.....................
		5	3	0.030	................t.....
		6	3	0.030	..t...................
		7	1	0.010	...........c..........
		8	1	0.010	..a...................
		9	1	0.010	.............t........
		10	1	0.010	...............t......
		11	1	0.010	.....................g
		12	1	0.010	....t.................
		Outgroup1	23	0.228	
	R	Total sequences	10106		CAATGCTGCAATCGTGCTACA
		Informative sequences	10098		10 20
		1	10070	99.644	....|....|....|....|.
		2	10	0.099	............a........
		3	4	0.040	.............t.......
		4	4	0.040	............t........
		5	3	0.030	t....................
		6	2	0.020	....t................
		7	2	0.020	...............t.....
		8	1	0.010	................t....
		9	1	0.010	................a....

			10	1	0.010	...c.................
			Outgroup1	8	0.079	
		P	Total sequences	10106		ATCACATTGGCACCCGCAATCCTG
			Informative sequences	10099		10 20
			1	9885	97.813	....|....|....|....|....
			2	158	1.563	....t...................
			3	19	0.188	.......................t
			4	9	0.089	........a...............
			5	8	0.079	....................t...
			6	8	0.079	....g...................
			7	5	0.049	..........t.............
			8	3	0.030	................t.......
			9	2	0.020	.........a..............
			10	1	0.010	.c......................
			11	1	0.010	.....................t..
			Outgroup1	7	0.069	
Thailand NIH	WH-NIC N	F	Total sequences	10106		CGTTTGGTGGACCCTCAGAT
			Informative sequences	10073		10 20
			1	9976	98.714	....|....|....|....|
			2	72	0.712	.....t..............
			3	14	0.139	......t.............
			4	3	0.030	.............t......
			5	2	0.020	..............c.....
			6	2	0.020	a...................
			7	2	0.020	............t.......
			8	1	0.010	t...................
			9	1	0.010	.........t..........
			Outgroup1	33	0.327	
		R	Total sequences	10106		AATGGAGAACGCAGTGGGG
			Informative sequences	10074		10
			1	10047	99.416	....|....|....|....
			2	8	0.079	.............t.....
			3	6	0.059	..................t

	4	3	0.030	.t.................
	5	3	0.030	...............t...
	6	3	0.030	..........a........
	7	3	0.030	................t..
	8	1	0.010	..........t........
	Outgroup1	32	0.317	
P	Total sequences	10106		CAACTGGCAGTAACCA
	Informative sequences	10075		10
	1	10066	99.604	....|....|....|.
	2	3	0.030	...t............
	3	2	0.020	.........t......
	4	2	0.020	..............a..
	5	1	0.010	..............t..
	6	1	0.010	............g...
	Outgroup1	31	0.307	

[a] The underlined letter within NIID_2019-nCoV_NR2 indicates a mismatched site to the PCR template, which should be G.

Table S13. Mismatches in the primer and probe targets of diagnostic PCR assays with Eta variant

<table>
<tr><th>Target gene</th><th>Institute</th><th>Primer name</th><th>Type</th><th>Group number</th><th>Variant count</th><th>Frequency (%)</th><th>Sequence (5’-3’)</th></tr>
<tr><td rowspan="17">E</td><td rowspan="17">Charité</td><td rowspan="17">E_Sarbeco</td><td rowspan="6">F</td><td>Total sequences</td><td>2041</td><td></td><td>ACAGGTACGTTAATAGTTAATAGCGT</td></tr>
<tr><td>Informative sequences</td><td>2040</td><td></td><td>10 20</td></tr>
<tr><td>1</td><td>2038</td><td>99.853</td><td>....|....|....|....|....|.</td></tr>
<tr><td>2</td><td>1</td><td>0.049</td><td>.t........................</td></tr>
<tr><td>3</td><td>1</td><td>0.049</td><td>...............a..........</td></tr>
<tr><td>Outgroup1</td><td>1</td><td>0.049</td><td></td></tr>
<tr><td rowspan="5">R</td><td>Total sequences</td><td>2041</td><td></td><td>TGTGTGCGTACTGCTGCAATAT</td></tr>
<tr><td>Informative sequences</td><td>1985</td><td></td><td>10 20</td></tr>
<tr><td>1</td><td>1984</td><td>97.207</td><td>....|....|....|....|..</td></tr>
<tr><td>2</td><td>1</td><td>0.049</td><td>....................g.</td></tr>
<tr><td>Outgroup1</td><td>56</td><td>2.744</td><td></td></tr>
<tr><td rowspan="4">P</td><td>Total sequences</td><td>2041</td><td></td><td>ACACTAGCCATCCTTACTGCGCTTCG</td></tr>
<tr><td>Informative sequences</td><td>2011</td><td></td><td>10 20</td></tr>
<tr><td>1</td><td>2011</td><td>98.530</td><td>....|....|....|....|....|.</td></tr>
<tr><td>Outgroup1</td><td>30</td><td>1.470</td><td></td></tr>
<tr><td rowspan="16">Orf1 RdRp</td><td rowspan="16">IP</td><td rowspan="16">nCoV_IP2</td><td rowspan="5">F</td><td>Total sequences</td><td>2041</td><td></td><td>ATGAGCTTAGTCCTGTTG</td></tr>
<tr><td>Informative sequences</td><td>2016</td><td></td><td>10</td></tr>
<tr><td>1</td><td>2010</td><td>98.481</td><td>....|....|....|...</td></tr>
<tr><td>2</td><td>6</td><td>0.294</td><td>...............c..</td></tr>
<tr><td>Outgroup1</td><td>25</td><td>1.225</td><td></td></tr>
<tr><td rowspan="8">R</td><td>Total sequences</td><td>2041</td><td></td><td>ACAACACAACAAAGGGAG</td></tr>
<tr><td>Informative sequences</td><td>2015</td><td></td><td>10</td></tr>
<tr><td>1</td><td>2005</td><td>98.236</td><td>....|....|....|...</td></tr>
<tr><td>2</td><td>5</td><td>0.245</td><td>....t.............</td></tr>
<tr><td>3</td><td>3</td><td>0.147</td><td>.t................</td></tr>
<tr><td>4</td><td>1</td><td>0.049</td><td>.........t........</td></tr>
<tr><td>5</td><td>1</td><td>0.049</td><td>.................t</td></tr>
<tr><td>Outgroup1</td><td>26</td><td>1.274</td><td></td></tr>
<tr><td rowspan="3">P</td><td>Total sequences</td><td>2041</td><td></td><td>AGATGTCTTGTGCTGCCGGTA</td></tr>
<tr><td>Informative sequences</td><td>2021</td><td></td><td>10 20</td></tr>
<tr><td>1</td><td>2021</td><td>99.020</td><td>....|....|....|....|.</td></tr>
</table>

			Outgroup1	20	0.980	
	nCoV_IP4	F	Total sequences	2041		GGTAACTGGTATGATTTCG
			Informative sequences	2039		10
			1	2039	99.902	....|....|....|....
			Outgroup1	2	0.098	
		R	Total sequences	2041		CCTATATTAACCTTGACCAG
			Informative sequences	2037		10 20
			1	2036	99.755	....|....|....|....|
			2	1	0.049	.................t..
			Outgroup1	4	0.196	
		P	Total sequences	2041		TCATACAAACCACGCCAGG
			Informative sequences	2038		10
			1	2031	99.510	....|....|....|....
			2	3	0.147	...........g.......
			3	3	0.147	...............t...
			4	1	0.049	.............a.....
			Outgroup1	3	0.147	
Charité	RdRp-SARS & RdRp-CoV-19	F	Total sequences	2041	RdRp-SARS	GTGARATGGTCATGTGTGGCGG
					RdRp-CoV-19	GTGAAATGGTCATGTGTGGCGG
			Informative sequences	2002		10 20
			1	2002	98.089	....|....|....|....|..
			Outgroup1	39	1.911	
		R	Total sequences	2041	RdRp-SARS	TATGCTAATAGTGTSTTTAACATRTG
					RdRp-CoV-19	TATGCTAATAGTGTTTTTAACATRTG
			Informative sequences	2002		10 20
			1	2002	98.089	....|....|....|....|...t|.
			Outgroup1	39	1.911	
		P	Total sequences	2041	RdRp-SARS	CCAGGTGGAACCTCATCAGGAGATGC
					RdRp-	CAGGTGGAACCTCATCAGGAGATGC

						CoV-19	
				Informative sequences	2002		10 20
				1	2002	98.089	....|....|....|....|....|.
				Outgroup1	39	1.911	
	China CDC	ORF1ab	F	Total sequences	2041		CCCTGTGGGTTTTACACTTAA
				Informative sequences	2025		10 20
				1	2024	99.167	....|....|....|....|.
				2	1	0.049	..............t......
				Outgroup1	16	0.784	
			R	Total sequences	2041		TCAGCTGATGCACAATCGT
				Informative sequences	2004		10
				1	2004	98.187	....|....|....|....
				Outgroup1	37	1.813	
			P	Total sequences	2041		CCGTCTGCGGTATGTGGAAAGGTTATGG
				Informative sequences	2007		10 20
				1	1994	97.697	....|....|....|....|....|...
				2	12	0.588	.t..........................
				3	1	0.049	.........t..................
				Outgroup1	34	1.666	
nsp14	HKU	ORF1b-nsp14	F	Total sequences	2041		TGGGGTTTTACAGGTAACCT
				Informative sequences	2029		10 20
				1	2026	99.265	....|....|....|....|
				2	2	0.098	....t...............
				3	1	0.049	..........t.........
				Outgroup1	12	0.588	
			R	Total sequences	2041		GAGTGCTTTGTTAAGCGRGTT
				Informative sequences	2022		10 20
				1	2020	98.971	....|....|....|..t.|.
				2	2	0.098	.........t.......t...
				Outgroup1	19	0.931	
			P	Total sequences	2041		TAGTTGTGATGCAATCATGACTAG
				Informative sequences	2022		10 20
				1	2022	99.069	....|....|....|....|....
				Outgroup1	19	0.931	

N		HKU-N	F	Total sequences	2041		TAATCAGACAAGGAACTGATTA
				Informative sequences	1929		10 20
				1	1925	94.317	....|....|....|....|..
				2	4	0.196	...............t......
				Outgroup1	5	0.245	
				Outgroup2	1	0.049	
				Excluded	106	5.194	
			R	Total sequences	2041		CATGGAAGTCACACCTTCG
				Informative sequences	1920		10
				1	1909	93.533	....|....|....|....
				2	5	0.245	..................a
				3	4	0.196	..................t
				4	2	0.098	.................t.
				Outgroup1	9	0.441	
				Excluded	112	5.488	
			P	Total sequences	2041		CCGCAAATTGCACAATTTGC
				Informative sequences	1924		10 20
				1	1915	93.827	....|....|....|....|
				2	4	0.196	................a...
				3	2	0.098	...........g........
				4	1	0.049	.....g..............
				5	1	0.049	..................t.
				6	1	0.049	..t.................
				Outgroup1	7	0.343	
				Outgroup2	3	0.147	
				Excluded	107	5.243	
	Japan NIID	NIID_2019-nCoV_2	F	Total sequences	2041		AAATTTTGGGGACCAGGAAC
				Informative sequences	1929		10 20
				1	1921	94.121	....|....|....|....|
				2	5	0.245	...............t....
				3	3	0.147	...................t
				Outgroup1	6	0.294	
				Excluded	106	5.194	
			R	Total sequences	2041		GTTGACCTACACAGCTGCCA[a]

<table>
<tr><td></td><td></td><td></td><td>Informative sequences</td><td>1919</td><td></td><td>10 20</td></tr>
<tr><td></td><td></td><td></td><td>1</td><td>1916</td><td>93.876</td><td>....|....|....g....|</td></tr>
<tr><td></td><td></td><td></td><td>2</td><td>2</td><td>0.098</td><td>.........t....g.....</td></tr>
<tr><td></td><td></td><td></td><td>3</td><td>1</td><td>0.049</td><td>.....a........g.....</td></tr>
<tr><td></td><td></td><td></td><td>Outgroup1</td><td>9</td><td>0.441</td><td></td></tr>
<tr><td></td><td></td><td></td><td>Outgroup2</td><td>1</td><td>0.049</td><td></td></tr>
<tr><td></td><td></td><td></td><td>Excluded</td><td>112</td><td>5.488</td><td></td></tr>
<tr><td></td><td></td><td>P</td><td>Total sequences</td><td>2041</td><td></td><td>ATGTCGCGCATTGGCATGGA</td></tr>
<tr><td></td><td></td><td></td><td>Informative sequences</td><td>1922</td><td></td><td>10 20</td></tr>
<tr><td></td><td></td><td></td><td>1</td><td>1921</td><td>94.121</td><td>....|....|....|....|</td></tr>
<tr><td></td><td></td><td></td><td>2</td><td>1</td><td>0.049</td><td>.......a............</td></tr>
<tr><td></td><td></td><td></td><td>Outgroup1</td><td>7</td><td>0.343</td><td></td></tr>
<tr><td></td><td></td><td></td><td>Outgroup2</td><td>2</td><td>0.098</td><td></td></tr>
<tr><td></td><td></td><td></td><td>Excluded</td><td>110</td><td>5.390</td><td></td></tr>
<tr><td>China CDC</td><td>N</td><td>F</td><td>Total sequences</td><td>2041</td><td></td><td>GGGGAACTTCTCCTGCTAGAAT</td></tr>
<tr><td></td><td></td><td></td><td>Informative sequences</td><td>2003</td><td></td><td>10 20</td></tr>
<tr><td></td><td></td><td></td><td>1</td><td>1970</td><td>96.521</td><td>....|.t..|....|....|..</td></tr>
<tr><td></td><td></td><td></td><td>2</td><td>22</td><td>1.078</td><td>......................</td></tr>
<tr><td></td><td></td><td></td><td>3</td><td>8</td><td>0.392</td><td>.....g................</td></tr>
<tr><td></td><td></td><td></td><td>4</td><td>3</td><td>0.147</td><td>aac...c...............</td></tr>
<tr><td></td><td></td><td></td><td>Outgroup1</td><td>30</td><td>1.470</td><td></td></tr>
<tr><td></td><td></td><td></td><td>Excluded</td><td>8</td><td>0.392</td><td></td></tr>
<tr><td></td><td></td><td>R</td><td>Total sequences</td><td>2041</td><td></td><td>CAGCTTGAGAGCAAAATGTCTG</td></tr>
<tr><td></td><td></td><td></td><td>Informative sequences</td><td>2002</td><td></td><td>10 20</td></tr>
<tr><td></td><td></td><td></td><td>1</td><td>1985</td><td>97.304</td><td>....|....|....|....|..</td></tr>
<tr><td></td><td></td><td></td><td>2</td><td>6</td><td>0.294</td><td>...........t..........</td></tr>
<tr><td></td><td></td><td></td><td>3</td><td>4</td><td>0.196</td><td>.................a....</td></tr>
<tr><td></td><td></td><td></td><td>4</td><td>3</td><td>0.147</td><td>..t...................</td></tr>
<tr><td></td><td></td><td></td><td>5</td><td>3</td><td>0.147</td><td>...................t..</td></tr>
<tr><td></td><td></td><td></td><td>6</td><td>1</td><td>0.049</td><td>.................t....</td></tr>
<tr><td></td><td></td><td></td><td>Outgroup1</td><td>30</td><td>1.471</td><td></td></tr>
<tr><td></td><td></td><td></td><td>Excluded</td><td>8</td><td>0.392</td><td></td></tr>
<tr><td></td><td></td><td>P</td><td>Total sequences</td><td>2041</td><td></td><td>TTGCTGCTGCTTGACAGATT</td></tr>
<tr><td></td><td></td><td></td><td>Informative sequences</td><td>2003</td><td></td><td>10 20</td></tr>
</table>

			1	2003	98.138	....|....|....|....|
			2	3	0.147	.....t..............
			3	1	0.049	..t.................
			Outgroup1	26	1.274	
			Excluded	8	0.392	
US CDC	2019-nCoV_N1	F	Total sequences	2041		GACCCCAAAATCAGCGAAAT
			Informative sequences	2024		10 20
			1	2024	99.167	....|....|....|....|
			Outgroup1	9	0.441	
			Excluded	8	0.392	
		R	Total sequences	2041		CAGATTCAACTGGCAGTAACCAGA
			Informative sequences	2024		10 20
			1	1941	95.100	....|....|....|....|....
			2	81	3.969	......................t.
			3	2	0.098	t.......................
			Outgroup1	9	0.441	
			Excluded	8	0.392	
		P	Total sequences	2041		ACCCCGCATTACGTTTGGTGGACC
			Informative sequences	2023		10 20
			1	2009	98.432	....|....|....|....|....
			2	12	0.588	....................c...
			3	1	0.049	.....a..................
			4	1	0.049	............a...........
			Outgroup1	10	0.490	
			Excluded	8	0.392	
	2019-nCoV_N2	F	Total sequences	2041		TTACAAACATTGGCCGCAAA
			Informative sequences	1927		10 20
			1	1921	94.121	....|....|....|....|
			2	3	0.147	.......t............
			3	1	0.049	..................g.
			4	1	0.049	...t................
			5	1	0.049	...............t....
			Outgroup1	6	0.294	
			Outgroup2	2	0.098	

		Excluded	106	5.194	
	R	Total sequences	2041		TTCTTCGGAATGTCGCGC
		Informative sequences	1922		10
		1	1828	89.564	....|....|....|...
		2	93	4.557	.....t............
		3	1	0.049	................a.
		Outgroup1	7	0.343	
		Outgroup2	2	0.098	
		Excluded	110	5.390	
	P	Total sequences	2041		ACAATTTGCCCCCAGCGCTTCAG
		Informative sequences	1924		10 20
		1	1914	93.778	....|....|....|....|...
		2	4	0.196	......a................
		3	2	0.098	g......................
		4	1	0.049	..............t........
		5	1	0.049	.......t...............
		6	1	0.049	............t..........
		7	1	0.049	.........t.............
		Outgroup1	7	0.343	
		Outgroup2	2	0.098	
		Excluded	108	5.292	
2019-nCoV_N3	F	Total sequences	2041		GGGAGCCTTGAATACACCAAAA
		Informative sequences	2022		10 20
		1	1996	97.795	....|....|....|...g|..
		2	10	0.490	....
		3	8	0.392	................t.....
		4	3	0.147	.........c............
		5	3	0.147	.a....................
		6	2	0.098	.........t............
		Outgroup1	11	0.539	
		Excluded	8	0.392	
	R	Total sequences	2041		CAATGCTGCAATCGTGCTACA
		Informative sequences	2033		10 20
		1	2009	98.432	....|....|....|....|.

			2	17	0.833	t....................
			3	7	0.343	............t........
			Excluded	8	0.392	
		P	Total sequences	2041		ATCACATTGGCACCCGCAATCCTG
			Informative sequences	2032		10 20
			1	2024	99.167	....|....|....|....|....
			2	5	0.245	....t...................
			3	3	0.147	................t.......
			Outgroup1	1	0.049	
			Excluded	8	0.392	
Thailand NIH	WH-NIC N	F	Total sequences	2041		CGTTTGGTGGACCCTCAGAT
			Informative sequences	2023		10 20
			1	2008	98.383	....|....|....|....|
			2	12	0.588	.........c..........
			3	2	0.098	...............t....
			4	1	0.049	.a..................
			Outgroup1	10	0.490	
			Excluded	8	0.392	
		R	Total sequences	2041		AATGGAGAACGCAGTGGGG
			Informative sequences	2024		10
			1	2023	99.118	....|....|....|....
			2	1	0.049	....t..............
			Outgroup1	9	0.441	
			Excluded	8	0.392	
		P	Total sequences	2041		CAACTGGCAGTAACCA
			Informative sequences	2024		10
			1	2024	99.167	....|....|....|.
			Outgroup1	9	0.441	
			Excluded	8	0.392	

[a] The underlined letter within NIID_2019-nCoV_NR2 indicates a mismatched site to the PCR template, which should be G.

Table S14. Mismatches in the primer and probe targets of diagnostic PCR assays with Kappa variant

Target gene	Institute	Primer name	Type	Group number	Variant count	Frequency(%)	Sequence (5’-3’)
E	Charité	E_Sarbeco	F	Total sequences	731		ACAGGTACGTTAATAGTTAATAGCGT
				Informative sequences	731		10 20
				1	731	100.000	....|....|....|....|....|.
			R	Total sequences	731		TGTGTGCGTACTGCTGCAATAT
				Informative sequences	731		10 20
				1	731	100.000	....|....|....|....|..
			P	Total sequences	731		ACACTAGCCATCCTTACTGCGCTTCG
				Informative sequences	731		10 20
				1	731	100.000	....|....|....|....|....|.
Orf1 RdRp	IP	nCoV_IP2	F	Total sequences	731		ATGAGCTTAGTCCTGTTG
				Informative sequences	729		10
				1	729	99.726	....|....|....|...
				Outgroup1	2	0.274	
			R	Total sequences	731		ACAACACAACAAAGGGAG
				Informative sequences	725		10
				1	724	99.042	....|....|....|...
				2	1	0.137	.........t........
				Outgroup1	6	0.821	
			P	Total sequences	731		AGATGTCTTGTGCTGCCGGTA
				Informative sequences	730		10 20
				1	726	99.316	....|....|....|....|.
				2	4	0.547	.............a.......
				Outgroup1	1	0.137	
		nCoV_IP4	F	Total sequences	731		GGTAACTGGTATGATTTCG
				Informative sequences	728		10
				1	728	99.590	....|....|....|....
				Outgroup1	3	0.410	
			R	Total sequences	731		CCTATATTAACCTTGACCAG
				Informative sequences	727		10 20
				1	725	99.179	....|....|....|....|
				2	1	0.137	.................t..

			3	1	0.137	...............t.....
			Outgroup1	4	0.547	
		P	Total sequences	731		TCATACAAACCACGCCAGG
			Informative sequences	727		10
			1	712	97.401	....|....|....|....
			2	12	1.642	...............t....
			3	2	0.274	................t...
			4	1	0.137	.................g..
			Outgroup1	4	0.547	
Charité	RdRp-SARS & RdRp-CoV-19	F	Total sequences	731	RdRp-SARS	GTGARATGGTCATGTGTGGCGG
					RdRp-CoV-19	GTGAAATGGTCATGTGTGGCGG
			Informative sequences	722		10 20
			1	720	98.495	....|....|....|....|..
			2	1	0.137	..............t........
			3	1	0.137	.....................a.
			Outgroup1	39	1.911	
		R	Total sequences	731	RdRp-SARS	TATGCTAATAGTGTSTTTAACATRTG
					RdRp-CoV-19	TATGCTAATAGTGTTTTTAACATRTG
			Informative sequences	727		10 20
			1	727	99.453	....|....|....|....|...t|.
			Outgroup1	4	0.547	
		P	Total sequences	731	RdRp-SARS	CCAGGTGGAACCTCATCAGGAGATGC
					RdRp-CoV-19	CAGGTGGAACCTCATCAGGAGATGC
			Informative sequences	727		10 20
			1	718	98.222	....|....|....|....|....|.
			2	4	0.547	.................t........
			3	1	0.137	...........t..............
			4	1	0.137	....................g.....

				Outgroup1	39	1.911	
	China CDC	ORF1ab	F	Total sequences	731		CCCTGTGGGTTTTACACTTAA
				Informative sequences	731		10 20
				1	731	100.000	....|....|....|....|.
			R	Total sequences	731		TCAGCTGATGCACAATCGT
				Informative sequences	725		10
				1	722	98.769	....|....|....|....
				2	1	0.137	....t..............
				3	1	0.137	.................a.
				4	1	0.137	.....a.............
				Outgroup1	37	1.813	
			P	Total sequences	731		CCGTCTGCGGTATGTGGAAAGGTTATGG
				Informative sequences	724		10 20
				1	719	98.358	....|....|....|....|....|...
				2	2	0.274	........a...................
				3	1	0.137	............a...............
				4	1	0.137	..........................t.
				5	1	0.137	.......t....................
				Outgroup1	34	1.666	
nsp14	HKU	ORF1b-nsp14	F	Total sequences	731		TGGGGTTTTACAGGTAACCT
				Informative sequences	730		10 20
				1	728	99.590	....|....|....|....|
				2	1	0.137	.................t..
				3	1	0.137	...........t........
				Outgroup1	1	0.137	
			R	Total sequences	731		GAGTGCTTTGTTAAGCGRGTT
				Informative sequences	727		10 20
				1	727	99.453	....|....|....|..t.|.
				Outgroup1	4	0.547	
			P	Total sequences	731		TAGTTGTGATGCAATCATGACTAG
				Informative sequences	727		10 20
				1	727	99.453	....|....|....|....|....
				Outgroup1	4	0.547	
N		HKU-N	F	Total sequences	731		TAATCAGACAAGGAACTGATTA

<table>
<tr><td></td><td></td><td></td><td>Informative sequences</td><td>725</td><td></td><td>10 20</td></tr>
<tr><td></td><td></td><td></td><td>1</td><td>725</td><td>99.179</td><td>....|....|....|....|..</td></tr>
<tr><td></td><td></td><td></td><td>Outgroup1</td><td>6</td><td>0.821</td><td></td></tr>
<tr><td></td><td></td><td>R</td><td>Total sequences</td><td>731</td><td></td><td>CATGGAAGTCACACCTTCG</td></tr>
<tr><td></td><td></td><td></td><td>Informative sequences</td><td>724</td><td></td><td>10</td></tr>
<tr><td></td><td></td><td></td><td>1</td><td>716</td><td>97.948</td><td>....|....|....|....</td></tr>
<tr><td></td><td></td><td></td><td>2</td><td>5</td><td>0.684</td><td>.................t.</td></tr>
<tr><td></td><td></td><td></td><td>3</td><td>3</td><td>0.410</td><td>..................t</td></tr>
<tr><td></td><td></td><td></td><td>Outgroup1</td><td>7</td><td>0.958</td><td></td></tr>
<tr><td></td><td></td><td>P</td><td>Total sequences</td><td>731</td><td></td><td>CCGCAAATTGCACAATTTGC</td></tr>
<tr><td></td><td></td><td></td><td>Informative sequences</td><td>724</td><td></td><td>10 20</td></tr>
<tr><td></td><td></td><td></td><td>1</td><td>724</td><td>99.042</td><td>....|....|....|....|</td></tr>
<tr><td></td><td></td><td></td><td>Outgroup1</td><td>7</td><td>0.958</td><td></td></tr>
<tr><td>Japan NIID</td><td>NIID_2019-nCoV_2</td><td>F</td><td>Total sequences</td><td>731</td><td></td><td>AAATTTTGGGGACCAGGAAC</td></tr>
<tr><td></td><td></td><td></td><td>Informative sequences</td><td>725</td><td></td><td>10 20</td></tr>
<tr><td></td><td></td><td></td><td>1</td><td>725</td><td>99.179</td><td>....|....|....|....|</td></tr>
<tr><td></td><td></td><td></td><td>Outgroup1</td><td>6</td><td>0.821</td><td></td></tr>
<tr><td></td><td></td><td>R</td><td>Total sequences</td><td>731</td><td></td><td>GTTGACCTACACAG<u>C</u>TGCCA[a]</td></tr>
<tr><td></td><td></td><td></td><td>Informative sequences</td><td>724</td><td></td><td>10 20</td></tr>
<tr><td></td><td></td><td></td><td>1</td><td>724</td><td>99.042</td><td>....|....|....g....|</td></tr>
<tr><td></td><td></td><td></td><td>Outgroup1</td><td>7</td><td>0.958</td><td></td></tr>
<tr><td></td><td></td><td>P</td><td>Total sequences</td><td>731</td><td></td><td>ATGTCGCGCATTGGCATGGA</td></tr>
<tr><td></td><td></td><td></td><td>Informative sequences</td><td>724</td><td></td><td>10 20</td></tr>
<tr><td></td><td></td><td></td><td>1</td><td>722</td><td>98.769</td><td>....|....|....|....|</td></tr>
<tr><td></td><td></td><td></td><td>2</td><td>1</td><td>0.137</td><td>............t.......</td></tr>
<tr><td></td><td></td><td></td><td>3</td><td>1</td><td>0.137</td><td>.....t..............</td></tr>
<tr><td></td><td></td><td></td><td>Outgroup1</td><td>7</td><td>0.958</td><td></td></tr>
<tr><td>China CDC</td><td>N</td><td>F</td><td>Total sequences</td><td>731</td><td></td><td>GGGGAACTTCTCCTGCTAGAAT</td></tr>
<tr><td></td><td></td><td></td><td>Informative sequences</td><td>714</td><td></td><td>10 20</td></tr>
<tr><td></td><td></td><td></td><td>1</td><td>706</td><td>96.580</td><td>t...|....|....|....|..</td></tr>
<tr><td></td><td></td><td></td><td>2</td><td>4</td><td>0.547</td><td>t.............t.......</td></tr>
<tr><td></td><td></td><td></td><td>3</td><td>3</td><td>0.410</td><td>aac...................</td></tr>
<tr><td></td><td></td><td></td><td>4</td><td>1</td><td>0.137</td><td>......................</td></tr>
<tr><td></td><td></td><td></td><td>Outgroup1</td><td>16</td><td>2.189</td><td></td></tr>
</table>

			Outgroup2	1	0.137	
		R	Total sequences	731		CAGCTTGAGAGCAAAATGTCTG
			Informative sequences	715		10 20
			1	712	97.401	....|....|....|....|..
			2	3	0.410	...................t..
			Outgroup1	16	2.189	
		P	Total sequences	731		TTGCTGCTGCTTGACAGATT
			Informative sequences	714		10 20
			1	713	97.538	....|....|....|....|
			2	1	0.137	..............t.....
			Outgroup1	17	2.326	
US CDC	2019-nCoV_N1	F	Total sequences	731		GACCCCAAAATCAGCGAAAT
			Informative sequences	729		10 20
			1	726	99.316	....|....|....|....|
			2	2	0.274	.............t......
			3	1	0.137	..........c.........
			Outgroup1	2	0.274	
		R	Total sequences	731		CAGATTCAACTGGCAGTAACCAGA
			Informative sequences	729		10 20
			1	725	99.179	....|....|....|....|....
			2	2	0.274	..t.....................
			3	1	0.137	.........t..............
			4	1	0.137	...............t........
			Outgroup1	2	0.274	
		P	Total sequences	731		ACCCCGCATTACGTTTGGTGGACC
			Informative sequences	729		10 20
			1	716	97.948	....|....|....|....|....
			2	7	0.958	.t......................
			3	2	0.274	.................t......
			4	2	0.274	...a....................
			5	1	0.137	...t....................
			6	1	0.137	............c...........
			Outgroup1	2	0.274	
	2019-	F	Total sequences	731		TTACAAACATTGGCCGCAAA

<table>
<tr><td>nCoV_N2</td><td></td><td>Informative sequences</td><td>725</td><td></td><td>10 20</td></tr>
<tr><td></td><td></td><td>1</td><td>722</td><td>98.769</td><td>....|....|....|....|</td></tr>
<tr><td></td><td></td><td>2</td><td>2</td><td>0.274</td><td>...t................</td></tr>
<tr><td></td><td></td><td>3</td><td>1</td><td>0.137</td><td>.........c..........</td></tr>
<tr><td></td><td></td><td>Outgroup1</td><td>6</td><td>0.821</td><td></td></tr>
<tr><td></td><td>R</td><td>Total sequences</td><td>731</td><td></td><td>TTCTTCGGAATGTCGCGC</td></tr>
<tr><td></td><td></td><td>Informative sequences</td><td>724</td><td></td><td>10</td></tr>
<tr><td></td><td></td><td>1</td><td>721</td><td>98.632</td><td>....|....|....|...</td></tr>
<tr><td></td><td></td><td>2</td><td>2</td><td>0.274</td><td>.....t............</td></tr>
<tr><td></td><td></td><td>3</td><td>1</td><td>0.137</td><td>..............t...</td></tr>
<tr><td></td><td></td><td>Outgroup1</td><td>7</td><td>0.958</td><td></td></tr>
<tr><td></td><td>P</td><td>Total sequences</td><td>731</td><td></td><td>ACAATTTGCCCCCAGCGCTTCAG</td></tr>
<tr><td></td><td></td><td>Informative sequences</td><td>724</td><td></td><td>10 20</td></tr>
<tr><td></td><td></td><td>1</td><td>721</td><td>98.632</td><td>....|....|....|....|...</td></tr>
<tr><td></td><td></td><td>2</td><td>3</td><td>0.410</td><td>..............a........</td></tr>
<tr><td></td><td></td><td>Outgroup1</td><td>7</td><td>0.958</td><td></td></tr>
<tr><td>2019-nCoV_N3</td><td>F</td><td>Total sequences</td><td>731</td><td></td><td>GGGAGCCTTGAATACACCAAAA</td></tr>
<tr><td></td><td></td><td>Informative sequences</td><td>730</td><td></td><td>10 20</td></tr>
<tr><td></td><td></td><td>1</td><td>723</td><td>98.906</td><td>....|....|....|....|..</td></tr>
<tr><td></td><td></td><td>2</td><td>6</td><td>0.821</td><td>..............t.......</td></tr>
<tr><td></td><td></td><td>3</td><td>1</td><td>0.137</td><td>.........t............</td></tr>
<tr><td></td><td></td><td>Outgroup1</td><td>1</td><td>0.137</td><td></td></tr>
<tr><td></td><td>R</td><td>Total sequences</td><td>731</td><td></td><td>CAATGCTGCAATCGTGCTACA</td></tr>
<tr><td></td><td></td><td>Informative sequences</td><td>730</td><td></td><td>10 20</td></tr>
<tr><td></td><td></td><td>1</td><td>723</td><td>98.906</td><td>....|....|....|....|.</td></tr>
<tr><td></td><td></td><td>2</td><td>3</td><td>0.410</td><td>.t...................</td></tr>
<tr><td></td><td></td><td>3</td><td>3</td><td>0.410</td><td>t....................</td></tr>
<tr><td></td><td></td><td>4</td><td>1</td><td>0.137</td><td>.......t.............</td></tr>
<tr><td></td><td></td><td>Outgroup1</td><td>1</td><td>0.137</td><td></td></tr>
<tr><td></td><td>P</td><td>Total sequences</td><td>731</td><td></td><td>ATCACATTGGCACCCGCAATCCTG</td></tr>
<tr><td></td><td></td><td>Informative sequences</td><td>723</td><td></td><td>10 20</td></tr>
<tr><td></td><td></td><td>1</td><td>723</td><td>98.906</td><td>....|....|....|....|....</td></tr>
<tr><td></td><td></td><td>2</td><td>5</td><td>0.684</td><td>....t...................</td></tr>
<tr><td></td><td></td><td>3</td><td>1</td><td>0.137</td><td>................t.......</td></tr>
</table>

			4	1	0.137	....................t...
			Outgroup1	1	0.137	
Thailand NIH	WH-NIC N	F	Total sequences	731		CGTTTGGTGGACCCTCAGAT
			Informative sequences	723		10 20
			1	723	98.906	....|....|....|....|
			2	2	0.274	.................t..
			3	2	0.274	......t.............
			4	1	0.137	.............a......
			5	1	0.137	...c................
			Outgroup1	2	0.274	
		R	Total sequences	731		AATGGAGAACGCAGTGGGG
			Informative sequences	728		10
			1	719	98.358	....|....|....|....
			2	9	1.231	..........a........
			Outgroup1	3	0.410	
		P	Total sequences	731		CAACTGGCAGTAACCA
			Informative sequences	729		10
			1	727	99.453	....|....|....|.
			2	1	0.137	...t............
			3	1	0.137	.........t......
			Outgroup1	2	0.274	

[a] The underlined letter within NIID_2019-nCoV_NR2 indicates a mismatched site to the PCR template, which should be G.

Table S15. Mismatches in the primer and probe targets of diagnostic PCR assays with Lambda variant

<table>
<tr><th>Target gene</th><th>Institute</th><th>Primer name</th><th>Type</th><th>Group number</th><th>Variant count</th><th>Frequency (%)</th><th>Sequence (5’-3’)</th></tr>
<tr><td>E</td><td>Charité</td><td>E_Sarbeco</td><td>F</td><td>Total sequences</td><td>628</td><td></td><td>ACAGGTACGTTAATAGTTAATAGCGT</td></tr>
<tr><td></td><td></td><td></td><td></td><td>Informative sequences</td><td>624</td><td></td><td>10 20</td></tr>
<tr><td></td><td></td><td></td><td></td><td>1</td><td>622</td><td>99.045</td><td>....|....|....|....|....|.</td></tr>
<tr><td></td><td></td><td></td><td></td><td>2</td><td>5</td><td>0.796</td><td>.t........................</td></tr>
<tr><td></td><td></td><td></td><td></td><td>Outgroup1</td><td>1</td><td>0.159</td><td></td></tr>
<tr><td></td><td></td><td></td><td>R</td><td>Total sequences</td><td>628</td><td></td><td>TGTGTGCGTACTGCTGCAATAT</td></tr>
<tr><td></td><td></td><td></td><td></td><td>Informative sequences</td><td></td><td></td><td>10 20</td></tr>
<tr><td></td><td></td><td></td><td></td><td>1</td><td>621</td><td>98.885</td><td>....|....|....|....|..</td></tr>
<tr><td></td><td></td><td></td><td></td><td>Outgroup1</td><td>7</td><td>1.115</td><td></td></tr>
<tr><td></td><td></td><td></td><td>P</td><td>Total sequences</td><td>628</td><td></td><td>ACACTAGCCATCCTTACTGCGCTTCG</td></tr>
<tr><td></td><td></td><td></td><td></td><td>Informative sequences</td><td>625</td><td></td><td>10 20</td></tr>
<tr><td></td><td></td><td></td><td></td><td>1</td><td>620</td><td>98.726</td><td>....|....|....|....|....|.</td></tr>
<tr><td></td><td></td><td></td><td></td><td>2</td><td>4</td><td>0.637</td><td>........t.................</td></tr>
<tr><td></td><td></td><td></td><td></td><td>3</td><td>1</td><td>0.159</td><td>..........c...............</td></tr>
<tr><td></td><td></td><td></td><td></td><td>Outgroup1</td><td>3</td><td>0.478</td><td></td></tr>
<tr><td>Orf1 RdRp</td><td>IP</td><td>nCoV_IP2</td><td>F</td><td>Total sequences</td><td>628</td><td></td><td>ATGAGCTTAGTCCTGTTG</td></tr>
<tr><td></td><td></td><td></td><td></td><td>Informative sequences</td><td>625</td><td></td><td>10</td></tr>
<tr><td></td><td></td><td></td><td></td><td>1</td><td>625</td><td>99.522</td><td>....|....|....|...</td></tr>
<tr><td></td><td></td><td></td><td></td><td>Outgroup1</td><td>3</td><td>0.478</td><td></td></tr>
<tr><td></td><td></td><td></td><td>R</td><td>Total sequences</td><td>628</td><td></td><td>ACAACACAACAAAGGGAG</td></tr>
<tr><td></td><td></td><td></td><td></td><td>Informative sequences</td><td>625</td><td></td><td>10</td></tr>
<tr><td></td><td></td><td></td><td></td><td>1</td><td>624</td><td>99.363</td><td>....|....|....|...</td></tr>
<tr><td></td><td></td><td></td><td></td><td>2</td><td>1</td><td>0.159</td><td>......t...........</td></tr>
<tr><td></td><td></td><td></td><td></td><td>Outgroup1</td><td>3</td><td>0.478</td><td></td></tr>
<tr><td></td><td></td><td></td><td>P</td><td>Total sequences</td><td>628</td><td></td><td>AGATGTCTTGTGCTGCCGGTA</td></tr>
<tr><td></td><td></td><td></td><td></td><td>Informative sequences</td><td>625</td><td></td><td>10 20</td></tr>
<tr><td></td><td></td><td></td><td></td><td>1</td><td>625</td><td>99.522</td><td>....|....|....|....|.</td></tr>
<tr><td></td><td></td><td></td><td></td><td>Outgroup1</td><td>3</td><td>0.478</td><td></td></tr>
<tr><td></td><td></td><td>nCoV_IP4</td><td>F</td><td>Total sequences</td><td>628</td><td></td><td>GGTAACTGGTATGATTTCG</td></tr>
<tr><td></td><td></td><td></td><td></td><td>Informative sequences</td><td>628</td><td></td><td>10</td></tr>
<tr><td></td><td></td><td></td><td></td><td>1</td><td>628</td><td>100.000</td><td>...................</td></tr>
</table>

<table>
<tr><td></td><td></td><td>R</td><td>Total sequences</td><td>628</td><td></td><td>CCTATATTAACCTTGACCAG</td></tr>
<tr><td></td><td></td><td></td><td>Informative sequences</td><td>628</td><td></td><td>10 20</td></tr>
<tr><td></td><td></td><td></td><td>1</td><td>624</td><td>99.363</td><td>....|....|....|....|</td></tr>
<tr><td></td><td></td><td></td><td>2</td><td>2</td><td>0.318</td><td>...........t........</td></tr>
<tr><td></td><td></td><td></td><td>3</td><td>1</td><td>0.159</td><td>...........a........</td></tr>
<tr><td></td><td></td><td></td><td>4</td><td>1</td><td>0.159</td><td>.................t..</td></tr>
<tr><td></td><td></td><td>P</td><td>Total sequences</td><td>628</td><td></td><td>TCATACAAACCACGCCAGG</td></tr>
<tr><td></td><td></td><td></td><td>Informative sequences</td><td>628</td><td></td><td>10</td></tr>
<tr><td></td><td></td><td></td><td>1</td><td>626</td><td>99.682</td><td>....|....|....|....</td></tr>
<tr><td></td><td></td><td></td><td>2</td><td>2</td><td>0.318</td><td>...............t...</td></tr>
<tr><td>Charité</td><td>RdRp-SARS & RdRp-CoV-19</td><td>F</td><td></td><td></td><td>RdRp-SARS</td><td>GTGARATGGTCATGTGTGGCGG</td></tr>
<tr><td></td><td></td><td></td><td>Total sequences</td><td>628</td><td>RdRp-CoV-19</td><td>GTGAAATGGTCATGTGTGGCGG</td></tr>
<tr><td></td><td></td><td></td><td>Informative sequences</td><td>621</td><td></td><td>10 20</td></tr>
<tr><td></td><td></td><td></td><td>1</td><td>621</td><td>98.885</td><td>....|....|....|....|..</td></tr>
<tr><td></td><td></td><td></td><td>Outgroup1</td><td>7</td><td>1.115</td><td></td></tr>
<tr><td></td><td></td><td>R</td><td></td><td></td><td>RdRp-SARS</td><td>TATGCTAATAGTGTSTTTAACATRTG</td></tr>
<tr><td></td><td></td><td></td><td>Total sequences</td><td>628</td><td>RdRp-CoV-19</td><td>TATGCTAATAGTGTTTTTAACATRTG</td></tr>
<tr><td></td><td></td><td></td><td>Informative sequences</td><td>621</td><td></td><td>10 20</td></tr>
<tr><td></td><td></td><td></td><td>1</td><td>621</td><td>98.885</td><td>....|....|....|....|...t|.</td></tr>
<tr><td></td><td></td><td></td><td>Outgroup1</td><td>7</td><td>1.115</td><td></td></tr>
<tr><td></td><td></td><td>P</td><td></td><td></td><td>RdRp-SARS</td><td>CCAGGTGGAACCTCATCAGGAGATGC</td></tr>
<tr><td></td><td></td><td></td><td>Total sequences</td><td>628</td><td>RdRp-CoV-19</td><td>CAGGTGGAACCTCATCAGGAGATGC</td></tr>
<tr><td></td><td></td><td></td><td>Informative sequences</td><td>621</td><td></td><td>10 20</td></tr>
<tr><td></td><td></td><td></td><td>1</td><td>621</td><td>98.885</td><td>....|....|....|....|....|.</td></tr>
<tr><td></td><td></td><td></td><td>Outgroup1</td><td>7</td><td>1.115</td><td></td></tr>
<tr><td>China CDC</td><td>ORF1ab</td><td>F</td><td>Total sequences</td><td>628</td><td></td><td>CCCTGTGGGTTTTACACTTAA</td></tr>
<tr><td></td><td></td><td></td><td>Informative sequences</td><td>625</td><td></td><td>10 20</td></tr>
<tr><td></td><td></td><td></td><td>1</td><td>625</td><td>99.522</td><td>....|....|....|....|.</td></tr>
<tr><td></td><td></td><td></td><td>Outgroup1</td><td>3</td><td>0.478</td><td></td></tr>
<tr><td></td><td></td><td>R</td><td>Total sequences</td><td>628</td><td></td><td>TCAGCTGATGCACAATCGT</td></tr>
<tr><td></td><td></td><td></td><td>Informative sequences</td><td>618</td><td></td><td>10</td></tr>
</table>

			1	618	98.408	....|....|....|....
			Outgroup1	10	1.592	
		P	Total sequences	628		CCGTCTGCGGTATGTGGAAAGGTTATGG
			Informative sequences	618		10 20
			1	618	98.408	....|....|....|....|....|...
			Outgroup1	10	1.592	
nsp14		F	Total sequences	628		TGGGGTTTTACAGGTAACCT
			Informative sequences	625		10 20
			1	625	99.522	....|....|....|....|
			Outgroup1	3	0.478	
	ORF1b-nsp14	R	Total sequences	628		GAGTGCTTTGTTAAGCGRGTT
			Informative sequences	615		10 20
			1	615	97.930	....|....|....|..t.|.
			2	1	0.159	..................tt..
			Outgroup1	12	1.911	
		P	Total sequences	628		TAGTTGTGATGCAATCATGACTAG
			Informative sequences	616		10 20
			1	616	98.089	....|....|....|....|....
			Outgroup1	12	1.911	
N	HKU	F	Total sequences	628		TAATCAGACAAGGAACTGATTA
			Informative sequences	627		10 20
			1	625	99.522	....|....|....|....|..
			2	1	0.159	...................c..
			3	1	0.159	.................a....
			Outgroup1	1	0.159	
	HKU-N	R	Total sequences	628		CATGGAAGTCACACCTTCG
			Informative sequences	627		10
			1	623	99.204	....|....|....|....
			2	4	0.637	..............t....
			Outgroup1	1	0.159	
		P	Total sequences	628		CCGCAAATTGCACAATTTGC
			Informative sequences	6		10 20
			1	627	99.841	....|....|....|....
			Outgroup1	1	0.159	

<table>
<tr><td rowspan="13">Japan NIID</td><td rowspan="13">NIID_2019-nCoV_2</td><td rowspan="4">F</td><td>Total sequences</td><td>628</td><td></td><td>AAATTTTGGGGACCAGGAAC</td></tr>
<tr><td>Informative sequences</td><td>627</td><td></td><td>10 20</td></tr>
<tr><td>1</td><td>627</td><td>99.841</td><td>....|....|....|....|</td></tr>
<tr><td>Outgroup1</td><td>1</td><td>0.159</td><td></td></tr>
<tr><td rowspan="5">R</td><td>Total sequences</td><td>628</td><td></td><td>GTTGACCTACACAG<u>C</u>TGCCA[a]</td></tr>
<tr><td>Informative sequences</td><td>626</td><td></td><td>10 20</td></tr>
<tr><td>1</td><td>622</td><td>99.045</td><td>....|....|....g....|</td></tr>
<tr><td>2</td><td>4</td><td>0.637</td><td>.........t....g.....</td></tr>
<tr><td>Outgroup1</td><td>2</td><td>0.318</td><td></td></tr>
<tr><td rowspan="4">P</td><td>Total sequences</td><td>628</td><td></td><td>ATGTCGCGCATTGGCATGGA</td></tr>
<tr><td>Informative sequences</td><td>625</td><td></td><td>10 20</td></tr>
<tr><td>1</td><td>625</td><td>99.522</td><td>....|....|....|....|</td></tr>
<tr><td>Outgroup1</td><td>3</td><td>0.478</td><td></td></tr>
<tr><td rowspan="17">China CDC</td><td rowspan="17">N</td><td rowspan="6">F</td><td>Total sequences</td><td>628</td><td></td><td>GGGGAACTTCTCCTGCTAGAAT</td></tr>
<tr><td>Informative sequences</td><td>624</td><td></td><td>10 20</td></tr>
<tr><td>1</td><td>622</td><td>99.045</td><td>aac.|....|....|....|..</td></tr>
<tr><td>2</td><td>1</td><td>0.159</td><td>aac...t...............</td></tr>
<tr><td>3</td><td>1</td><td>0.159</td><td>aac...........t.......</td></tr>
<tr><td>Outgroup1</td><td>4</td><td>0.637</td><td></td></tr>
<tr><td rowspan="6">R</td><td>Total sequences</td><td>628</td><td></td><td>CAGCTTGAGAGCAAAATGTCTG</td></tr>
<tr><td>Informative sequences</td><td>626</td><td></td><td>10 20</td></tr>
<tr><td>1</td><td>624</td><td>99.363</td><td>....|....|....|....|..</td></tr>
<tr><td>2</td><td>1</td><td>0.159</td><td>.................a....</td></tr>
<tr><td>3</td><td>1</td><td>0.159</td><td>....................a.</td></tr>
<tr><td>Outgroup1</td><td>2</td><td>0.318</td><td></td></tr>
<tr><td rowspan="5">P</td><td>Total sequences</td><td>628</td><td></td><td>TTGCTGCTGCTTGACAGATT</td></tr>
<tr><td>Informative sequences</td><td>627</td><td></td><td>10 20</td></tr>
<tr><td>1</td><td>625</td><td>99.522</td><td>....|....|....|....|</td></tr>
<tr><td>2</td><td>2</td><td>0.318</td><td></td></tr>
<tr><td>Outgroup1</td><td>1</td><td>0.159</td><td></td></tr>
<tr><td rowspan="4">US CDC</td><td rowspan="4">2019-nCoV_N1</td><td rowspan="4">F</td><td>Total sequences</td><td>628</td><td></td><td>GACCCCAAAATCAGCGAAAT</td></tr>
<tr><td>Informative sequences</td><td>625</td><td></td><td>10 20</td></tr>
<tr><td>1</td><td>621</td><td>98.885</td><td>....|....|....|....|</td></tr>
<tr><td>2</td><td>2</td><td>0.318</td><td>.................g..</td></tr>
</table>

		3	2	0.318	..........c.........
		Outgroup1	3	0.478	
	R	Total sequences	628		CAGATTCAACTGGCAGTAACCAGA
		Informative sequences	625		10 20
		1	624	99.363	....|....|....|....|....
		2	1	0.159	.....c..................
		Outgroup1	3	0.478	
	P	Total sequences	628		ACCCCGCATTACGTTTGGTGGACC
		Informative sequences	624		10 20
		1	613	97.611	..t.|....|....|....|....
		2	7	1.115	.t......................
		3	3	0.478	..c.....................
		4	1	0.159	...t....................
		Outgroup1	4	0.637	
2019-nCoV_N2	F	Total sequences	628		TTACAAACATTGGCCGCAAA
		Informative sequences	627		10 20
		1	625	99.522	....|....|....|....|
		2	1	0.159	...t................
		3	1	0.159	c...................
		Outgroup1	1	0.159	
	R	Total sequences	628		TTCTTCGGAATGTCGCGC
		Informative sequences	625		10
		1	625	99.522	....|....|....|...
		Outgroup1	3	0.478	
	P	Total sequences	628		ACAATTTGCCCCCAGCGCTTCAG
		Informative sequences	626		10 20
		1	626	99.682	....|....|....|....|...
		Outgroup1	2	0.318	
2019-nCoV_N3	F	Total sequences	628		GGGAGCCTTGAATACACCAAAA
		Informative sequences	625		10 20
		1	624	99.363	....|....|....|....|..
		2	1	0.159	t.....................
		Outgroup1	2	0.318	

				Outgroup2	1	0.159	
			R	Total sequences	628		CAATGCTGCAATCGTGCTACA
				Informative sequences	627		10 20
				1	627	99.841	....|....|....|....|.
				Outgroup1	1	0.159	
			P	Total sequences	628		ATCACATTGGCACCCGCAATCCTG
				Informative sequences	628		10 20
				1	624	99.363	....|....|....|....|....
				2	3	0.478	..t......................
				3	1	0.159	........................t
			F	Total sequences	628		CGTTTGGTGGACCCTCAGAT
				Informative sequences	625		10 20
				1	624	99.363	....|....|....|....|
				2	1	0.159	.............a......
				Outgroup1	3	0.478	
	Thailand NIH	WH-NIC N	R	Total sequences	628		AATGGAGAACGCAGTGGGG
				Informative sequences	625		10
				1	625	99.522	....|....|....|....
				Outgroup1	3	0.478	
			P	Total sequences	628		CAACTGGCAGTAACCA
				Informative sequences	625		10
				1	625	99.522	....|....|....|.
				Outgroup1	3	0.478	

[a] The underlined letter within NIID_2019-nCoV_NR2 indicates a mismatched site to the PCR template, which should be G.

Table S16. Mismatches in the primer and probe targets of diagnostic PCR assays with Mu variant

Target gene	Institute	Primer name	Type	Group number	Variant count	Frequency (%)	Sequence (5'-3')
E	Charité	E_Sarbeco	F	Total sequences	1506		ACAGGTACGTTAATAGTTAATAGCGT
				Informative sequences	1487		10 20
				1	1487	98.738	....|....|....|....|....|.
				Outgroup1	19	1.262	
			R	Total sequences	628		TGTGTGCGTACTGCTGCAATAT
				Informative sequences			10 20

					1	1485	98.606	....|....|....|....|..
					2	1	0.066	......t...............
					Outgroup1	20	1.328	
				P	Total sequences	1506		ACACTAGCCATCCTTACTGCGCTTCG
					Informative sequences	1488		10 20
					1	1486	98.672	....|....|....|....|....|.
					2	2	0.133	........t.................
					Outgroup1	18	1.195	
Orf1	RdRp	IP	nCoV_IP2	F	Total sequences	1506		ATGAGCTTAGTCCTGTTG
					Informative sequences	1505		10
					1	1503	99.801	....|....|....|...
					2	1	0.066	................a.
					3	1	0.066	..............a...
					Outgroup1	1	0.066	
				R	Total sequences	1506		ACAACACAACAAAGGGAG
					Informative sequences	1506		10
					1	1506	100.000	....|....|....|...
				P	Total sequences	1506		AGATGTCTTGTGCTGCCGGTA
					Informative sequences	1503		10 20
					1	1501	99.668	....|....|....|....|.
					2	2	0.133	.a...................
					Outgroup1	3	0.199	
			nCoV_IP4	F	Total sequences	1506		GGTAACTGGTATGATTTCG
					Informative sequences	1505		10
					1	1505	99.934	....|....|....|....
					Outgroup1	1	0.066	
				R	Total sequences	1506		CCTATATTAACCTTGACCAG
					Informative sequences	1502		10 20
					1	1495	99.270	....|....|....|....|
					2	5	0.332	...........t........
					3	2	0.133	.................t..
					Outgroup1	4	0.266	
				P	Total sequences	1506		TCATACAAACCACGCCAGG
					Informative sequences	1506		10

			1	1477	98.074	....|....|....|....
			2	23	1.527	...........g.......
			3	3	0.199	................t..
			4	2	0.133	..................a
			5	1	0.066	...............t...
Charité	RdRp-SARS & RdRp-CoV-19	F	Total sequences	1506	RdRp-SARS	GTGARATGGTCATGTGTGGCGG
					RdRp-CoV-19	GTGAAATGGTCATGTGTGGCGG
			Informative sequences	1505		10 20
			1	1504	99.867	....|....|....|....|..
			2	1	0.066	..........t...........
			Outgroup1	1	0.066	
		R	Total sequences	1506	RdRp-SARS	TATGCTAATAGTGTSTTTAACATRTG
					RdRp-CoV-19	TATGCTAATAGTGTTTTTAACATRTG
			Informative sequences	1506		10 20
			1	1506	100.000	....|....|....|....|...t|.
		P	Total sequences	1505	RdRp-SARS	CCAGGTGGAACCTCATCAGGAGATGC
					RdRp-CoV-19	CAGGTGGAACCTCATCAGGAGATGC
			Informative sequences	1505		10 20
			1	1505	99.934	....|....|....|....|....|.
			Outgroup1	1	0.066	
China CDC	ORF1ab	F	Total sequences	1505		CCCTGTGGGTTTTACACTTAA
			Informative sequences	1505		10 20
			1	1504	99.867	....|....|....|....|.
			2	1	0.066	..............t......
			Outgroup1	1	0.066	
		R	Total sequences	1505		TCAGCTGATGCACAATCGT
			Informative sequences	1474		10
			1	1469	97.543	....|....|....|....
			2	4	0.266	.................a.
			3	1	0.066	................t..
			Outgroup1	32	2.125	

		P	Total sequences	1506		CCGTCTGCGGTATGTGGAAAGGTTATGG
			Informative sequences	1482		10 20
			1	1481	98.340	....|....|....|....|....|...
			2	1	0.066	.......t....................
			Outgroup1	23	1.527	
			Outgroup2	1	0.066	
nsp14	HKU ORF1b-nsp14	F	Total sequences	1506		TGGGGTTTTACAGGTAACCT
			Informative sequences	1504		10 20
			1	1502	99.734	....|....|....|....|
			2	2	0.133	..........t.........
			Outgroup1	2	0.133	
		R	Total sequences	1506		GAGTGCTTTGTTAAGCGRGTT
			Informative sequences	1450		10 20
			1	1447	96.082	....|....|....|..t.|.
			2	3	0.199	.............g...t...
			Outgroup1	56	3.718	
		P	Total sequences	1506		TAGTTGTGATGCAATCATGACTAG
			Informative sequences	1449		10 20
			1	1446	96.016	....|....|....|....|....
			2	3	0.199	..........t.............
			Outgroup1	57	3.785	
N	HKU-N	F	Total sequences	1506		TAATCAGACAAGGAACTGATT
			Informative sequences	1501		10 20
			1	1501	99.668	....|....|....|....|..
			Outgroup1	5	0.332	
		R	Total sequences	1506		CATGGAAGTCACACCTTCG
			Informative sequences	1496		10
			1	1490	98.938	....|....|....|....
			2	2	0.133	..................t
			3	2	0.133	.............t.....
			4	1	0.066	....a..............
			5	1	0.066	..............t....
			Outgroup1	9	0.598	
			Outgroup2	1	0.066	

<table>
<tr><td></td><td></td><td>P</td><td>Total sequences</td><td>1506</td><td></td><td>CCGCAAATTGCACAATTTGC</td></tr>
<tr><td></td><td></td><td></td><td>Informative sequences</td><td>1496</td><td></td><td>10 20</td></tr>
<tr><td></td><td></td><td></td><td>1</td><td>1496</td><td>99.336</td><td>....|....|....|....|</td></tr>
<tr><td></td><td></td><td></td><td>Outgroup1</td><td>10</td><td>0.664</td><td></td></tr>
<tr><td>Japan NIID</td><td>NIID_2019-nCoV_2</td><td>F</td><td>Total sequences</td><td>1506</td><td></td><td>AAATTTTGGGGACCAGGAAC</td></tr>
<tr><td></td><td></td><td></td><td>Informative sequences</td><td>1499</td><td></td><td>10 20</td></tr>
<tr><td></td><td></td><td></td><td>1</td><td>1499</td><td>99.535</td><td>....|....|....|....|</td></tr>
<tr><td></td><td></td><td></td><td>Outgroup1</td><td>7</td><td>0.465</td><td></td></tr>
<tr><td></td><td></td><td>R</td><td>Total sequences</td><td>1506</td><td></td><td>GTTGACCTACACAGCTGCCA[a]</td></tr>
<tr><td></td><td></td><td></td><td>Informative sequences</td><td>1494</td><td></td><td>10 20</td></tr>
<tr><td></td><td></td><td></td><td>1</td><td>1490</td><td>98.938</td><td>....|....|....g....|</td></tr>
<tr><td></td><td></td><td></td><td>2</td><td>3</td><td>0.199</td><td>....................</td></tr>
<tr><td></td><td></td><td></td><td>3</td><td>1</td><td>0.066</td><td>............t.......</td></tr>
<tr><td></td><td></td><td></td><td>Outgroup1</td><td>11</td><td>0.730</td><td>...........t........</td></tr>
<tr><td></td><td></td><td></td><td>Excluded</td><td>1</td><td>0.066</td><td></td></tr>
<tr><td></td><td></td><td>P</td><td>Total sequences</td><td>1506</td><td></td><td>ATGTCGCGCATTGGCATGGA</td></tr>
<tr><td></td><td></td><td></td><td>Informative sequences</td><td>1495</td><td></td><td>10 20</td></tr>
<tr><td></td><td></td><td></td><td>1</td><td>1474</td><td>97.875</td><td>....|....|....|....|</td></tr>
<tr><td></td><td></td><td></td><td>2</td><td>20</td><td>1.328</td><td>.....t..............</td></tr>
<tr><td></td><td></td><td></td><td>3</td><td>1</td><td>0.066</td><td>..................a.</td></tr>
<tr><td></td><td></td><td></td><td>Outgroup1</td><td>11</td><td>0.730</td><td></td></tr>
<tr><td>China CDC</td><td>N</td><td>F</td><td>Total sequences</td><td>1506</td><td></td><td>GGGGAACTTCTCCTGCTAGAAT</td></tr>
<tr><td></td><td></td><td></td><td>Informative sequences</td><td>1491</td><td></td><td>10 20</td></tr>
<tr><td></td><td></td><td></td><td>1</td><td>1486</td><td>98.672</td><td>....|.t..|....|....|..</td></tr>
<tr><td></td><td></td><td></td><td>2</td><td>3</td><td>0.199</td><td>......................</td></tr>
<tr><td></td><td></td><td></td><td>3</td><td>1</td><td>0.066</td><td>..........c...........</td></tr>
<tr><td></td><td></td><td></td><td>4</td><td>1</td><td>0.066</td><td>..................t...</td></tr>
<tr><td></td><td></td><td></td><td>Outgroup1</td><td>15</td><td>0.996</td><td></td></tr>
<tr><td></td><td></td><td>R</td><td>Total sequences</td><td>1506</td><td></td><td>CAGCTTGAGAGCAAAATGTCTG</td></tr>
<tr><td></td><td></td><td></td><td>Informative sequences</td><td>1504</td><td></td><td>10 20</td></tr>
<tr><td></td><td></td><td></td><td>1</td><td>1475</td><td>97.942</td><td>....|....|....|....|..</td></tr>
<tr><td></td><td></td><td></td><td>2</td><td>10</td><td>0.664</td><td>....................a.</td></tr>
<tr><td></td><td></td><td></td><td>3</td><td>3</td><td>0.199</td><td>...a..................</td></tr>
<tr><td></td><td></td><td></td><td>4</td><td>3</td><td>0.199</td><td>..........a...........</td></tr>
</table>

			5	2	0.133	...t.................
			Outgroup1	2	0.318	
		P	Total sequences	1506		TTGCTGCTGCTTGACAGATT
			Informative sequences	1496		10 20
			1	1495	99.270	....|....|....|....|
			2	1	0.066	...........c........
			Outgroup1	10	0.664	
US CDC	2019-nCoV_N1	F	Total sequences	1506		GACCCCAAAATCAGCGAAAT
			Informative sequences	1501		10 20
			1	1498	99.469	....|....|....|....|
			2	2	0.133	....t...............
			3	1	0.066	a...................
			Outgroup1	5	0.332	
		R	Total sequences	1506		CAGATTCAACTGGCAGTAACCAGA
			Informative sequences	1598		10 20
			1	1487	98.738	....|....|....|....|....
			2	8	0.531	.........t..............
			3	2	0.133	..t.....................
			4	1	0.066	...g....................
			Outgroup1	8	0.531	
		P	Total sequences	1506		ACCCCGCATTACGTTTGGTGGACC
			Informative sequences	1500		10 20
			1	1484	98.539	....|....|....|....|....
			2	5	0.332	....
			3	4	0.266	..t.....................
			4	2	0.133	.a......................
			5	2	0.133	................t.......
			6	1	0.066	...........t............
			7	1	0.066	...t....................
			8	1	0.066	......t.................
			Outgroup1	6	0.398	
	2019-nCoV_N2	F	Total sequences	1506		TTACAAACATTGGCCGCAAA
			Informative sequences	1496		10 20
			1	1493	99.137	....|....|....|....|

			2	1	0.066	........g...........
			3	1	0.066	.......t............
			4	1	0.066	...t................
			Outgroup1	10	0.664	
		R	Total sequences	1506		TTCTTCGGAATGTCGCGC
			Informative sequences	1495		10
			1	1475	97.942	....|....|....|...
			2	20	1.328	..............t...
			Outgroup1	11	0.730	
		P	Total sequences	1506		ACAATTTGCCCCCAGCGCTTCAG
			Informative sequences	1497		10 20
			1	1484	98.539	....|....|....|....|...
			2	11	0.730	............t..........
			3	2	0.133	..............a........
			Outgroup1	9	0.598	
	2019-nCoV_N3	F	Total sequences	1506		GGGAGCCTTGAATACACCAAAA
			Informative sequences	1498		10 20
			1	1496	99.336	....|....|....|....|..
			2	1	0.066	..............t.......
			3	1	0.066	................t.....
			Outgroup1	8	0.531	
		R	Total sequences	1506		CAATGCTGCAATCGTGCTACA
			Informative sequences	1504		10 20
			1	1501	99.668	....|....|....|....|.
			2	3	0.199	.....t...............
			Outgroup1	2	0.133	
		P	Total sequences	1506		ATCACATTGGCACCCGCAATCCTG
			Informative sequences	1498		10 20
			1	1494	99.203	....|....|....|....|....
			2	3	0.199	..t.....................
			3	1	0.066	.......................a
			Outgroup1	8	0.531	
Thailand NIH	WH-NIC N	F	Total sequences	1506		CGTTTGGTGGACCCTCAGAT
			Informative sequences	1500		10 20

<table>
<tr><td></td><td></td><td></td><td></td><td>1</td><td>1492</td><td>99.070</td><td>....|....|....|....|</td></tr>
<tr><td></td><td></td><td></td><td></td><td>2</td><td>2</td><td>0.133</td><td>.................t..</td></tr>
<tr><td></td><td></td><td></td><td></td><td>3</td><td>2</td><td>0.133</td><td>.....t..............</td></tr>
<tr><td></td><td></td><td></td><td></td><td>4</td><td>2</td><td>0.133</td><td>t...................</td></tr>
<tr><td></td><td></td><td></td><td></td><td>5</td><td>1</td><td>0.066</td><td>..................g.</td></tr>
<tr><td></td><td></td><td></td><td></td><td>6</td><td>1</td><td>0.066</td><td>......t.............</td></tr>
<tr><td></td><td></td><td></td><td></td><td>Outgroup1</td><td>6</td><td>0.398</td><td></td></tr>
<tr><td></td><td></td><td></td><td rowspan="6">R</td><td>Total sequences</td><td>1506</td><td></td><td>AATGGAGAACGCAGTGGGG</td></tr>
<tr><td></td><td></td><td></td><td>Informative sequences</td><td>1494</td><td></td><td>10</td></tr>
<tr><td></td><td></td><td></td><td>1</td><td>1491</td><td>99.004</td><td>....|....|....|....</td></tr>
<tr><td></td><td></td><td></td><td>2</td><td>2</td><td>0.133</td><td>...............t...</td></tr>
<tr><td></td><td></td><td></td><td>3</td><td>1</td><td>0.066</td><td>.............t.....</td></tr>
<tr><td></td><td></td><td></td><td>Outgroup1</td><td>12</td><td>0.797</td><td></td></tr>
<tr><td></td><td></td><td></td><td rowspan="5">P</td><td>Total sequences</td><td>1506</td><td></td><td>CAACTGGCAGTAACCA</td></tr>
<tr><td></td><td></td><td></td><td>Informative sequences</td><td>1498</td><td></td><td>10</td></tr>
<tr><td></td><td></td><td></td><td>1</td><td>1490</td><td>98.938</td><td>....|....|....|.</td></tr>
<tr><td></td><td></td><td></td><td>2</td><td>8</td><td>0.531</td><td>...t............</td></tr>
<tr><td></td><td></td><td></td><td>Outgroup1</td><td>8</td><td>0.531</td><td></td></tr>
</table>

[a] The underlined letter within NIID_2019-nCoV_NR2 indicates a mismatched site to the PCR template, which should be G.

Table S17. Mismatches in the primer and probe targets of diagnostic PCR assays with Omicron variant

<table>
<tr><th>Target gene</th><th>Institute</th><th>Primer name</th><th>Type</th><th>Group number</th><th>Variant count</th><th>Frequency</th><th>Sequence（5’-3’）</th></tr>
<tr><td rowspan="10">E</td><td rowspan="10">Charité</td><td rowspan="10">E_Sarbeco</td><td rowspan="9">F</td><td>Total sequences</td><td>4588</td><td></td><td>ACAGGTACGTTAATAGTTAATAGCGT</td></tr>
<tr><td>Informative sequences</td><td>4560</td><td></td><td>10 20</td></tr>
<tr><td>1</td><td>4546</td><td>99.69298</td><td>.t..|....|....|....|....|.</td></tr>
<tr><td>2</td><td>10</td><td>0.219298</td><td>..........................</td></tr>
<tr><td>3</td><td>4</td><td>0.087719</td><td>.y........................</td></tr>
<tr><td>Outgroup1</td><td>24</td><td>0.526316</td><td></td></tr>
<tr><td>Outgroup2</td><td>2</td><td>0.04386</td><td></td></tr>
<tr><td>Outgroup3</td><td>1</td><td>0.02193</td><td></td></tr>
<tr><td>Outgroup4</td><td>1</td><td>0.02193</td><td></td></tr>
<tr><td>R</td><td>Total sequences</td><td>4588</td><td></td><td>TGTGTGCGTACTGCTGCAATAT</td></tr>
</table>

					Informative sequences	4346		10 20
					1	4345	94.704	....|....|....|....|..
					2	1	0.022	..g...................
					Outgroup1	123	2.681	
					Outgroup2	112	2.441	
					Outgroup3	1	0.022	
					Outgroup4	1	0.022	
					Outgroup5	1	0.022	
					Outgroup6	1	0.022	
					Outgroup7	1	0.022	
					Outgroup8	1	0.022	
				P	Total sequences	4588		ACACTAGCCATCCTTACTGCGCTTCG
					Informative sequences	4456		10 20
					1	4456	98.672	....|....|....|....|....|.
					Outgroup1	88	1.918	
					Outgroup2	29	0.632	
					Outgroup3	3	0.065	
					Outgroup4	2	0.044	
					Outgroup5	2	0.044	
					Outgroup6	1	0.022	
					Outgroup7	1	0.022	
					Outgroup8	1	0.022	
					Outgroup9	1	0.022	
					Outgroup10	1	0.022	
					Outgroup11	1	0.022	
					Outgroup12	1	0.022	
					Outgroup13	1	0.022	
Orf1	RdRp	IP	nCoV_IP2	F	Total sequences	4588		ATGAGCTTAGTCCTGTTG
					Informative sequences	4586		10
					1	4586	99.956	....|....|....|...
					Outgroup1	1	0.022	
					Outgroup2	1	0.022	
				R	Total sequences	4588		ACAACACAACAAAGGGAG
					Informative sequences	4586		10

<table>
<tr><td></td><td></td><td></td><td>1</td><td>4565</td><td>99.499</td><td>....|....|....|...</td></tr>
<tr><td></td><td></td><td></td><td>2</td><td>10</td><td>0.218</td><td>.................t</td></tr>
<tr><td></td><td></td><td></td><td>3</td><td>8</td><td>0.174</td><td>.t................</td></tr>
<tr><td></td><td></td><td></td><td>4</td><td>3</td><td>0.065</td><td>......t...........</td></tr>
<tr><td></td><td></td><td></td><td>Outgroup1</td><td>1</td><td>0.022</td><td></td></tr>
<tr><td></td><td></td><td></td><td>Outgroup2</td><td>1</td><td>0.022</td><td></td></tr>
<tr><td></td><td></td><td></td><td>Total sequences</td><td>4588</td><td></td><td>AGATGTCTTGTGCTGCCGGTA</td></tr>
<tr><td></td><td></td><td></td><td>Informative sequences</td><td>4582</td><td></td><td>10 20</td></tr>
<tr><td></td><td></td><td></td><td>1</td><td>4582</td><td>99.869</td><td>....|....|....|....|.</td></tr>
<tr><td></td><td></td><td>P</td><td>Outgroup1</td><td>3</td><td>0.065</td><td></td></tr>
<tr><td></td><td></td><td></td><td>Outgroup2</td><td>1</td><td>0.022</td><td></td></tr>
<tr><td></td><td></td><td></td><td>Outgroup3</td><td>1</td><td>0.022</td><td></td></tr>
<tr><td></td><td></td><td></td><td>Outgroup4</td><td>1</td><td>0.022</td><td></td></tr>
<tr><td></td><td></td><td></td><td>Total sequences</td><td>4588</td><td></td><td>GGTAACTGGTATGATTTCG</td></tr>
<tr><td></td><td></td><td>F</td><td>Informative sequences</td><td>4586</td><td></td><td>10</td></tr>
<tr><td></td><td></td><td></td><td>1</td><td>4586</td><td>99.956</td><td>....|....|....|....</td></tr>
<tr><td></td><td></td><td></td><td>Outgroup1</td><td>2</td><td>0.044</td><td></td></tr>
<tr><td></td><td></td><td></td><td>Total sequences</td><td>4588</td><td></td><td>CCTATATTAACCTTGACCAG</td></tr>
<tr><td></td><td></td><td></td><td>Informative sequences</td><td>4582</td><td></td><td>10 20</td></tr>
<tr><td></td><td></td><td></td><td>1</td><td>4572</td><td>99.651</td><td>....|....|....|....|</td></tr>
<tr><td></td><td></td><td></td><td>2</td><td>7</td><td>0.153</td><td>.................t..</td></tr>
<tr><td></td><td></td><td>R</td><td>3</td><td>3</td><td>0.065</td><td>.....g..............</td></tr>
<tr><td></td><td>nCoV_IP4</td><td></td><td>Outgroup1</td><td>4</td><td>0.087</td><td></td></tr>
<tr><td></td><td></td><td></td><td>Outgroup2</td><td>1</td><td>0.022</td><td></td></tr>
<tr><td></td><td></td><td></td><td>Outgroup3</td><td>1</td><td>0.022</td><td></td></tr>
<tr><td></td><td></td><td></td><td>Total sequences</td><td>4588</td><td></td><td>TCATACAAACCACGCCAGG</td></tr>
<tr><td></td><td></td><td></td><td>Informative sequences</td><td>4587</td><td></td><td>10</td></tr>
<tr><td></td><td></td><td></td><td>1</td><td>4564</td><td>99.477</td><td>....|....|....|....</td></tr>
<tr><td></td><td></td><td></td><td>2</td><td>15</td><td>0.327</td><td>..g................</td></tr>
<tr><td></td><td></td><td>P</td><td>3</td><td>5</td><td>0.109</td><td>..............t....</td></tr>
<tr><td></td><td></td><td></td><td>4</td><td>2</td><td>0.044</td><td>...............t...</td></tr>
<tr><td></td><td></td><td></td><td>5</td><td>1</td><td>0.022</td><td>.............t.....</td></tr>
<tr><td></td><td></td><td></td><td>Outgroup1</td><td>1</td><td>0.022</td><td></td></tr>
<tr><td>Charité</td><td>RdRp-SARS</td><td>F</td><td>Total sequences</td><td>4588</td><td>RdRp-</td><td>GTGARATGGTCATGTGTGGCGG</td></tr>
</table>

& RdRp-CoV-19			SARS RdRp-CoV-19	GTGAAATGGTCATGTGTGGCGG
	Informative sequences	4548		10 20
	1	4539	98.932	....|....|....|....|..
	2	5	0.109	....................r.
	3	3	0.065	....................a.
	4	1	0.022	.c....................
	Outgroup1	20	0.436	
	Outgroup2	4	0.087	
	Outgroup3	4	0.087	
	Outgroup4	2	0.044	
	Outgroup5	1	0.022	
	Outgroup6	1	0.022	
	Outgroup7	1	0.022	
	Outgroup8	1	0.022	
	Outgroup9	1	0.022	
	Outgroup10	1	0.022	
	Outgroup11	1	0.022	
	Outgroup12	1	0.022	
	Outgroup13	1	0.022	
	Outgroup14	1	0.022	
	Total sequences	4588	RdRp-SARS	TATGCTAATAGTGTSTTTAACATRTG
			RdRp-CoV-19	TATGCTAATAGTGTTTTTAACATRTG
	Informative sequences	4559		10 20
R	1	4418	96.295	....|....|....|....|...t|.
	2	98	2.136	................a.........
	3	40	0.872	................w.........
	4	3	0.065	.....c..........a.........
	5	1	0.022	....y...........a.........
	Outgroup1	9	0.196	

			Outgroup2	8	0.174	
			Outgroup3	2	0.044	
			Outgroup4	1	0.022	
			Outgroup5	1	0.022	
			Outgroup6	1	0.022	
			Outgroup7	1	0.022	
			Outgroup8	1	0.022	
			Outgroup9	1	0.022	
			Outgroup10	1	0.022	
			Outgroup11	1	0.022	
		P	Total sequences	4588	RdRp-SARS	CCAGGTGGAACCTCATCAGGAGATGC
					RdRp-CoV-19	CAGGTGGAACCTCATCAGGAGATGC
			Informative sequences	4560		10 20
			1	4557	99.324	....|....|....|....|....|.
			2	3	0.065	........a.................
			Outgroup1	20	0.436	
			Outgroup2	1	0.022	
			Outgroup3	1	0.022	
			Outgroup4	1	0.022	
			Outgroup5	1	0.022	
			Outgroup6	1	0.022	
			Outgroup7	1	0.022	
			Outgroup8	1	0.022	
			Outgroup9	1	0.022	
China CDC	ORF1ab	F	Total sequences	4588		CCCTGTGGGTTTTACACTTAA
			Informative sequences	4586		10 20
			1	4586	99.956	....|....|....|....|.
			2	2	0.044	..............t......
		R	Total sequences	4588		TCAGCTGATGCACAATCGT
			Informative sequences	1474		10
			1	4574	99.695	....|....|....|....
			Outgroup1	11	0.240	

<table>
<tr><td></td><td></td><td></td><td></td><td>Outgroup2</td><td>2</td><td>0.044</td><td></td></tr>
<tr><td></td><td></td><td></td><td></td><td>Outgroup3</td><td>1</td><td>0.022</td><td></td></tr>
<tr><td></td><td></td><td></td><td>P</td><td>Total sequences</td><td>4588</td><td></td><td>CCGTCTGCGGTATGTGGAAAGGTTATGG</td></tr>
<tr><td></td><td></td><td></td><td></td><td>Informative sequences</td><td>4568</td><td></td><td>10 20</td></tr>
<tr><td></td><td></td><td></td><td></td><td>1</td><td>4568</td><td>99.564</td><td>....|....|....|....|....|...</td></tr>
<tr><td></td><td></td><td></td><td></td><td>2</td><td>3</td><td>0.065</td><td>.t..........................</td></tr>
<tr><td></td><td></td><td></td><td></td><td>3</td><td>2</td><td>0.044</td><td>.y..........................</td></tr>
<tr><td></td><td></td><td></td><td></td><td>4</td><td>1</td><td>0.022</td><td>....y.......................</td></tr>
<tr><td></td><td></td><td></td><td></td><td>Outgroup1</td><td>7</td><td>0.153</td><td></td></tr>
<tr><td></td><td></td><td></td><td></td><td>Outgroup2</td><td>1</td><td>0.022</td><td></td></tr>
<tr><td></td><td></td><td></td><td></td><td>Outgroup3</td><td>1</td><td>0.022</td><td></td></tr>
<tr><td></td><td></td><td></td><td></td><td>Outgroup4</td><td>1</td><td>0.022</td><td></td></tr>
<tr><td></td><td></td><td></td><td></td><td>Outgroup5</td><td>1</td><td>0.022</td><td></td></tr>
<tr><td></td><td></td><td></td><td></td><td>Outgroup6</td><td>1</td><td>0.022</td><td></td></tr>
<tr><td></td><td></td><td></td><td></td><td>Outgroup7</td><td>1</td><td>0.022</td><td></td></tr>
<tr><td></td><td></td><td></td><td></td><td>Outgroup8</td><td>1</td><td>0.022</td><td></td></tr>
<tr><td>nsp14</td><td>HKU</td><td>ORF1b-nsp14</td><td>F</td><td>Total sequences</td><td>4588</td><td></td><td>TGGGGTTTTACAGGTAACCT</td></tr>
<tr><td></td><td></td><td></td><td></td><td>Informative sequences</td><td>4587</td><td></td><td>10 20</td></tr>
<tr><td></td><td></td><td></td><td></td><td>1</td><td>4585</td><td>99.935</td><td>....|....|....|....|</td></tr>
<tr><td></td><td></td><td></td><td></td><td>2</td><td>1</td><td>0.022</td><td>..........t.........</td></tr>
<tr><td></td><td></td><td></td><td></td><td>Outgroup1</td><td>1</td><td>0.022</td><td></td></tr>
<tr><td></td><td></td><td></td><td></td><td>Outgroup2</td><td>1</td><td>0.022</td><td></td></tr>
<tr><td></td><td></td><td></td><td>R</td><td>Total sequences</td><td>4588</td><td></td><td>GAGTGCTTTGTTAAGCGRGTT</td></tr>
<tr><td></td><td></td><td></td><td></td><td>Informative sequences</td><td>4513</td><td></td><td>10 20</td></tr>
<tr><td></td><td></td><td></td><td></td><td>1</td><td>4513</td><td>98.365</td><td>....|....|....|..t.|.</td></tr>
<tr><td></td><td></td><td></td><td></td><td>Outgroup1</td><td>73</td><td>1.591</td><td></td></tr>
<tr><td></td><td></td><td></td><td></td><td>Outgroup2</td><td>1</td><td>0.022</td><td></td></tr>
<tr><td></td><td></td><td></td><td></td><td>Outgroup3</td><td>1</td><td>0.022</td><td></td></tr>
<tr><td></td><td></td><td></td><td>P</td><td>Total sequences</td><td>4588</td><td></td><td>TAGTTGTGATGCAATCATGACTAG</td></tr>
<tr><td></td><td></td><td></td><td></td><td>Informative sequences</td><td>4514</td><td></td><td>10 20</td></tr>
<tr><td></td><td></td><td></td><td></td><td>1</td><td>4512</td><td>98.344</td><td>....|....|....|....|....</td></tr>
<tr><td></td><td></td><td></td><td></td><td>2</td><td>1</td><td>0.022</td><td>...............m........</td></tr>
<tr><td></td><td></td><td></td><td></td><td>3</td><td>1</td><td>0.022</td><td>..................a.....</td></tr>
<tr><td></td><td></td><td></td><td></td><td>Outgroup1</td><td>71</td><td>1.548</td><td></td></tr>
</table>

			Outgroup2	2	0.044	
			Outgroup3	1	0.022	
N	HKU-N	F	Total sequences	4588		TAATCAGACAAGGAACTGATT
			Informative sequences	4582		10 20
			1	4582	99.869	....|....|....|....|..
			Outgroup1	2	0.044	
			Outgroup2	1	0.022	
			Excluded	3	0.065	
		R	Total sequences	4588		CATGGAAGTCACACCTTCG
			Informative sequences	4582		10
			1	4574	99.695	....|....|....|....
			2	2	0.044	..................t
			3	1	0.022	.................t.
			Outgroup1	5	0.109	
			Excluded	6	0.131	
		P	Total sequences	4588		CCGCAAATTGCACAATTTGC
			Informative sequences	4582		10 20
			1	4582	99.869	....|....|....|....|
			Outgroup1	2	0.044	
			Excluded	4	0.087	
	Japan NIID	NIID_2019-nCoV_2	F	Total sequences	4588		AAATTTTGGGGACCAGGAAC
				Informative sequences	4579		10 20
				1	4578	99.782	....|....|....|....|
				2	1	0.022	...............c....
				Outgroup1	3	0.065	
				Outgroup2	2	0.044	
				Outgroup3	1	0.022	
				Excluded	3	0.065	
			R	Total sequences	4588		GTTGACCTACACAGCTGCCA[a]
				Informative sequences	4573		10 20
				1	4573	99.673	....|....|....g....|
				Outgroup1	7	0.153	
				Outgroup2	2	0.044	
				Excluded	6	0.131	

		P	Total sequences	1506		ATGTCGCGCATTGGCATGGA
			Informative sequences	1495		10 20
			1	4576	99.738	....|....|....|....|
			2	1	0.022	.....t..............
			Outgroup1	5	0.109	
			Excluded	6	0.131	
China CDC	N	F	Total sequences	4588		GGGGAACTTCTCCTGCTAGAAT
			Informative sequences	4577		10 20
			1	4563	99.455	....|....|....|....|..
			2	5	0.109	wrs...................
			3	5	0.109	ggg...................
			4	1	0.022	...............t......
			5	1	0.022	..................t...
			6	1	0.022	wr....................
			7	1	0.022	agg...................
			Outgroup1	5	0.109	
			Outgroup2	2	0.044	
			Outgroup3	1	0.022	
			Excluded	3	0.065	
		R	Total sequences	4588		CAGCTTGAGAGCAAAATGTCTG
			Informative sequences	4576		10 20
			1	4576	99.738	....|....|....|....|..
			Outgroup1	6	0.131	
			Outgroup2	1	0.022	
			Outgroup3	1	0.022	
			Outgroup4	1	0.022	
			Excluded	3	0.065	
		P	Total sequences	4588		TTGCTGCTGCTTGACAGATT
			Informative sequences	4577		10 20
			1	4570	99.608	....|....|....|....|
			2	5	0.109	........a...........
			3	1	0.022	......t.............
			4	1	0.022	.y..................

			Outgroup1	4	0.087	
			Outgroup2	2	0.044	
			Outgroup3	1	0.022	
			Outgroup4	1	0.022	
			Excluded	3	0.065	
US CDC	2019-nCoV_N1	F	Total sequences	4588		GACCCCAAAATCAGCGAAAT
			Informative sequences	4582		10 20
			1	4582	99.869	....|....|....|....|
			Outgroup1	6	0.131	
		R	Total sequences	4588		CAGATTCAACTGGCAGTAACCAGA
			Informative sequences	4575		10 20
			1	4570	99.608	....
			2	4	0.087	.........t..............
			3	1	0.022	.......t................
			Outgroup1	13	0.283	
		P	Total sequences	4588		ACCCCGCATTACGTTTGGTGGACC
			Informative sequences	4575		10 20
			1	4541	98.976	..t.....................
			2	21	0.458	....
			3	7	0.153	..y.....................
			4	2	0.044	..t....................t
			5	1	0.022	..t.........k...........
			6	1	0.022	..ty....................
			7	1	0.022	..t...a.................
			Outgroup1	6	0.022	
			Outgroup2	4	0.131	
			Outgroup3	3	0.087	
			Outgroup4	1	0.065	
	2019-nCoV_N2	F	Total sequences	4588		TTACAAACATTGGCCGCAAA
			Informative sequences	4582		10 20
			1	4578	99.782	....|....|....|....|
			2	3	0.065	.......t............
			3	1	0.022	...t................
			Outgroup1	3	0.065	

		Excluded	3	0.065	
	R	Total sequences	4588		TTCTTCGGAATGTCGCGC
		Informative sequences	4577		10
		1	4576	99.738	....|....|....|...
		2	1	0.022	..............t...
		Outgroup1	5	0.109	
		Excluded	6	0.131	
	P	Total sequences	4588		ACAATTTGCCCCCAGCGCTTCAG
		Informative sequences	4577		10 20
		1	4576	99.738	....|....|....|....|...
		2	1	0.022	............t..........
		Outgroup1	3	0.065	
		Outgroup2	3	0.065	
		Outgroup3	2	0.044	
		Excluded	3	0.065	
2019-nCoV_N3	F	Total sequences	4588		GGGAGCCTTGAATACACCAAAA
		Informative sequences	4566		10 20
		1	4560	99.390	....|....|....|....|..
		2	4	0.087	..............t.......
		3	1	0.022	t.....................
		4	1	0.022	...g..................
		Outgroup1	12	0.262	
		Outgroup2	5	0.109	
		Outgroup3	2	0.044	
		Excluded	3	0.065	
	R	Total sequences	4588		CAATGCTGCAATCGTGCTACA
		Informative sequences	4579		10 20
		1	4579	99.804	....|....|....|....|.
		Outgroup1	5	0.109	
		Outgroup2	1	0.022	
		Excluded	3	0.065	
	P	Total sequences	4588		ATCACATTGGCACCCGCAATCCTG
		Informative sequences	4574		10 20

			1	4571	99.629	....|....|....|....|....
			2	2	0.044	....t...................
			3	1	0.022	t.......................
			Outgroup1	5	0.109	
			Outgroup2	5	0.109	
			Outgroup3	1	0.022	
			Excluded	3	0.065	
Thailand NIH	WH-NIC N	F	Total sequences	4588		CGTTTGGTGGACCCTCAGAT
			Informative sequences	4574		10 20
			1	4572	99.651	....|....|....|....|
			2	2	0.044	............t.......
			3	1	0.022	.k..................
			Outgroup1	13	0.283	
		R	Total sequences	4588		AATGGAGAACGCAGTGGGG
			Informative sequences	14		10
			1	13	0.283	....|....|....|....
			2	1	0.022	.....t.....a.......
			Outgroup1	4483	97.711	
			Outgroup2	45	0.981	
			Outgroup3	19	0.414	
			Outgroup4	11	0.240	
			Outgroup5	2	0.044	
			Outgroup6	2	0.044	
			Outgroup7	1	0.022	
			Outgroup8	1	0.022	
			Outgroup9	1	0.022	
			Outgroup10	1	0.022	
			Outgroup11	1	0.022	
			Outgroup12	1	0.022	
			Outgroup13	1	0.022	
			Outgroup14	1	0.022	
			Outgroup15	1	0.022	
			Outgroup16	1	0.022	

	Outgroup17	1	0.022	
	Outgroup18	1	0.022	
P	Total sequences	4588		CAACTGGCAGTAACCA
	Informative sequences	4574		10
	1	4570	99.608	....|....|....|.
	2	4	0.087	...t............
	3	1	0.022	.t..............
	Outgroup1	13	0.283	

[a] The underlined letter within NIID_2019-nCoV_NR2 indicates a mismatched site to the PCR template, which should be G.

Table S18. Performance summary of the primers

Target gene		Institute	Primer name	Standard plasmids	TaqMan		SYBR Green		Significant mismatch with variants					
					Sensitivity	Specificity	Sensitivity	Specificity	Delta	Eta	Kappa	Lambda	Mu	Omicron
E		Charité	E_Sarbeco	Excluded								F[a], P[b]		
ORF1	RdRp	IP	nCoV_IP2	Success	High	Low	Moderate	Low			P[b]			
			nCoV_IP4	Fail					R[c]		P[b]		P[b]	
		Charité	RdRp-SARS	Fail					F[a], R[c]	R[c]	P[b], R[c]	R[c]	R[c]	R[c]
			RdRp-CoV-19	Fail										
		China CDC	ORF1ab	Success	Low	High	Moderate	High		P[b]				
	nsp14	HKU	ORF1b-nsp14	Success	Low	High	High	Low	R[c]	R[c]	R[c]	R[c]	R[c]	R[c]
N			HKU-N	Success	High	High	High	Low	R[c]		R[c]	R[c]		
		Japan NIID	NIID_2019-nCoV_2	Success	Low	High	High	Low				R[c]	P[b]	
		China CDC	N	Success	High	Low	High	Low	F[a]	F[a]	F[a]	F[a]	F[a], R[c]	
		US CDC	2019-nCoV_N1	Success	High	Low	High	Moderate	P[b]	P[b], R[c]	P[b]	P[b]	R[c]	P[b]
			2019-nCoV_N2	Fail						R[c]			P[b], R[c]	
			2019-nCoV_N3	Success	High	Low	High	Moderate	P[b]	F[a], R[c]	F[a], P[b]			
		Thailand NIH	WH-NIC N	Success	High	Low	High	Low	F[a]	F[a]	R[c]		P[b]	

[a] F: forward primer; [b] P: probe; [c] R: reverse primer.

Reference

Giulietti A, Overbergh L, Valckx D, Decallonne B, Bouillon R, Mathieu C (2001) An overview of real-time quantitative PCR: applications to quantify cytokine gene expression. Methods 25(4):386-401 doi: 10.1006/meth.2001.1261

Gudnason H, Dufva M, Bang DD, Wolff A (2007) Comparison of multiple DNA dyes for real-time PCR: effects of dye concentration and sequence composition on DNA amplification and melting temperature. Nucleic Acids Res 35(19):e127 doi: 10.1093/nar/gkm671

Hanna SE, Connor CJ, Wang HH (2005) Real-time polymerase chain reaction for the food microbiologist: technologies, applications, and limitations. J Food Sci 70(3):R49-R53 doi: 10.1111/j.1365-2621.2005.tb07149.x

Kubista M, Andrade JM, Bengtsson M, Forootan A, Jonak J, Lind K, Sindelka R, Sjoback R, Sjogreen B, Strombom L, Stahlberg A, Zoric N (2006) The real-time polymerase chain reaction. Mol Asp Med 27(2-3):95-125 doi: 10.1016/j.mam.2005.12.007

Okubara PA, Schroeder KL, Paulitz TC (2005) Real-time polymerase chain reaction: applications to studies on soilborne pathogens. Can J Plant Sci 27(3):300-313 doi: 10.1080/07060660509507229

Vogels CBF, Brito AF, Wyllie AL, Fauver JR, Ott IM, Kalinich CC, Petrone ME, Casanovas-Massana A, Catherine Muenker M, Moore AJ, Klein J, Lu P, Lu-Culligan A, Jiang X, Kim DJ, Kudo E, Mao T, Moriyama M, Oh JE, Park A, Silva J, Song E, Takahashi T, Taura M, Tokuyama M, Venkataraman A, Weizman OE, Wong P, Yang Y, Cheemarla NR, White EB, Lapidus S, Earnest R, Geng B, Vijayakumar P, Odio C, Fournier J, Bermejo S, Farhadian S, Dela Cruz CS, Iwasaki A, Ko AI, Landry ML, Foxman EF, Grubaugh ND (2020) Analytical sensitivity and efficiency comparisons of SARS-CoV-2 RT-qPCR primer-probe sets. Nat Microbiol 5(10):1299-1305 doi: 10.1038/s41564-020-0761-6
